# Phenotypic variability and genome-wide association studies in potato (*Solanum tuberosum* L.) for phosphorus efficiency

**DOI:** 10.1186/s12870-025-07018-3

**Published:** 2025-08-02

**Authors:** Mousumi Hazarika, Klaus J. Dehmer, Ralf Uptmoor, Mareike Kavka, Julian Kirchgesser, Doerte Harpke, Silvia Bachmann-Pfabe

**Affiliations:** 1 Satellite Collections North, Gross Luesewitz Potato Collections (GLKS), Genebank Department, Leibniz Institute of Plant Genetics and Crop Plant Research (IPK), Parkweg 3a, Sanitz OT, Gross Luesewitz 18190 Germany; 2 Agronomy, Faculty for Agricultural and Environmental Sciences, University of Rostock, Justus-von-Liebig-Weg 6, Rostock, 18059 Germany; 3 Experimental Taxonomy (ETX), Genebank Department, Leibniz Institute of Plant Genetics and Crop Plant Research (IPK), Corrensstrasse 3, Seeland, Gatersleben, OT D-06466 Germany; 4 Plant Nutrition and Soil Science, Faculty for Agriculture and Food Sciences, University of Applied Sciences Neubrandenburg, Brodaer Straße 2, Neubrandenburg, 17033 Germany

**Keywords:** Diversity, Genebank, Genetic resources, Nutrient efficiency, Pot experiment

## Abstract

**Background:**

Potatoes require phosphorus (P) for growth, tuber production and starch quality, but their small and shallow root system limits the nutrient acquisition. This results in excessive use of mineral P fertilisers, leading to environmental and economic concerns due to resource depletion. Identifying potato genotypes with high P efficiency and understanding the underlying genes responsible are crucial for molecular breeding and crop improvement. The present study aims at exploring the phenotypic and genotypic variation among potato genotypes from Gross Luesewitz Potato Collections (GLKS) and identifying markers significantly associated with P efficiency.

**Results:**

Phenotypic characterisation of a diversity set of 200 potato accessions for their response to P deprivation in a pot experiment showed a significant variance regarding biomass production, root length, plants height and P efficiency between the genotypes, with moderate to high heritability for these traits. Shoot biomass decreased by 66% on average under low P, while the root biomass decreased by 36% on average. The extent of the reduction was genotype specific, with some genotypes exhibiting higher root biomass and longer root length under low P than in control conditions. Outstanding genotypes were identified such as GLKS 11,578 (Kristall) with a dense and shallow root system or GLKS 10,591 (Tiger) with a long and extensive root system. Genome-wide association analysis identified 27 unique significant marker-trait associations including 13 associated with biomass, 3 with plant height and 11 with P efficiency, with a majority of them related to P utilization efficiency (7) and shoot biomass (11).

**Conclusion:**

Our research is one the firsts to present a genome-wide association study in potatoes for P efficiency. Our study highlighted significant phenotypic variation among the genotypes, while promising targets for improving P efficiency traits in potato through genomic selection and marker-assisted breeding.

**Supplementary Information:**

The online version contains supplementary material available at 10.1186/s12870-025-07018-3.

## Background

Potato is the fourth most widely produced crop globally and the most important non-grain food crop [1]. With the increasing interest in the use of agricultural crops for non-food application, potatoes play an important role as raw material for the starch industry, besides serving as a staple food in many cultures [2]. According to a report published by Vilpoux et al. [3] potatoes contribute about 3.9 million tons to the global starch production of 88.1 million tons, accounting for 4.4% of the industry raw material after corn (74.7%), tapioca (13.7%) and wheat (7.7%). The reason for its high popularity is its molecular structure and organization, which makes potato starch unique from that of other botanical origins. Compared to starch from other plant species, it is relatively pure, being constituted mainly of medium sized starch granules and scarce amounts of fat and protein [4]. Another unique feature of potato starch is the presence of an extremely high degree of phosphate monoesters linked to the amylopectin branches of the starch polymer [5, 6]. Studies conducted by Noda et al. [6] showed that enhancing the starch phosphate content resulted in significant increases in starch qualities like swelling power, viscosity and gelatinization temperatures. Thus, an adequate amount of phosphorus (P) is not only essential for rapid canopy development, tuber set, tuber yield, and nutritional quality, but is also critical for enhancing potato starch qualities and synthesis [7]. Some prior field trials have confirmed that mineral P fertilization increased tuber starch content and marketable tuber yield compared to the unfertilized control [8].

Even though P has been proven to be an essential macronutrient required for plant’s growth and metabolism, as it is a part of various biomolecules (e.g. DNA, ATP, and phospholipids), its uptake from fertilizer by the plant is limited [9, 10]. Soil P exists in various chemical forms including inorganic (Pi) and organic P [11]. It accounts for about 35 − 70% and 30 − 65%, respectively [11, 12] out of which plants can take up P primarily as Pi. In spite of being abundantly present in the soil in various forms, P is scarcely available to plants due to its extremely low diffusion rate and fixation by various minerals [12].

The sparse and shallow root system of potatoes makes it difficult to acquire P from the deeper soil layers [13]. Previous studies have shown that potato plants require more P for optimized yield performance than most other crops [14]. As a consequence, higher amounts of P fertilizers are applied to potatoes to increase P availability compared to other crops [15]. However, even though P fertilizer is applied, potato roots can acquire only about 30% of the applied P, while the rest is fixed by mineral soil compounds and microbes [16, 17] or is lost via surface run-off, contributing to eutrophication and hypoxia of water bodies [18, 19]. Moreover, non-renewable rock phosphate reserves, which are the main source of mineral P fertilizer, could be exhausted in the near future as the rate of use of P fertilizer is continuously increasing [20].

Studies have shown that plants use various strategies to mitigate P scarcity in the soil like increased root growth, formation of fine lateral roots, enhanced secretion of inorganic and organic compounds like protons, anions, sugars, organic acids, and enzymes [21, 22]. Crop genotypes with well-developed root systems with larger surface areas and higher root elongation rates exhibit a higher P acquisition efficiency [23] resulting in higher shoot biomass [24]. Previous experiments with potato showed increased root development with strong relation to P uptake under P deficiency. However, this was highly genotype dependent [25, 26].

At the molecular level, increased P uptake efficiency is realized by the enhanced expression of genes encoding for P transporters [27]. Phosphatases, which can significantly contribute to releasing plant-available P from organic compounds in the soil, have also been reported to be secreted more under P deficiency [28, 29]. An increased gene expression of *purple acid phosphatases* (*PAPs*) and the phosphate transporter *PHT1* was seen under P deficiency [9]. Grierson et al. [30] reported a number of P-deficiency responsive genes, which were related to root hair initiation and development in *Arabidopsis*. Raghothama [31] suggested that more than 100 genes are involved in plant´s response to low P. Among them are various transcription factors like *MYB*, *G-box* genes, *MADs box* transcription factors, and *ANR1* [32]. Cho and Cosgrove [33] elucidated that the genes that are related to biosynthesis of hormones like auxin and ethylene (*ACC oxidase*,* ACC oxidase*,* methionine synthase*,* S-adenosylmethionine synthetase* and *ctr1*) also contribute to efficient P uptake by modifying root architecture [34]. Under P deficiency, organic acid exudates like malate and citrate, which take part in Pi mobilization, were also reported to be secreted in very high amounts [9]. In response to P deficiency, many plants have been shown to adjust their metabolic rates and utilize alternative metabolic pathways as a defense strategy to conserve the internal Pi pool, in addition to increasing P uptake from soil [9]. For instance, alternative glycolytic reactions can bypass Pi or ATP-requiring steps of glycolysis under Pi starvation by using pyrophosphate [9].

Thus, besides adapting P fertilizer management practices including proper dosage and placement at the right time for optimal plant-available soil-P concentrations during critical growth stages, it is crucial to develop cultivars with higher P efficiency. This can be achieved by unveiling adaptation strategies at the molecular level, i.e. by dissecting the genetic architecture of the desired traits and by identifying genes and quantitative trait loci (QTL) which play a role in P scarcity tolerance and enhanced nutrient uptake. New molecular markers are required in breeding programs using marker assisted selection (MAS) approaches to develop crops well-adapted to the low availability of P in soil. Genome wide association studies (GWAS), which utilize a panel of diverse genotypes and next-generation sequencing (NGS) techniques to identify single nucleotide polymorphisms (SNPs), have been proven to be a powerful tool to identify QTL for higher stress tolerance in response to P scarcity [35]. In this regard, exploring the genetic variation in cultivated potatoes and their wild relatives can be crucial for developing adapted potato varieties. With respect to P efficiency traits, associations have been detected in maize, rice, and soybean [36]. Hammond et al. [37] used the SolCAP Infinium array to identify genes related to P deficiency during early growth in potato. To date, only few studies using whole genome sequencing and GWAS have been conducted in potato to improve tolerance to P deficiency.

Thus, keeping the importance of P uptake efficiency and its genetic architecture in mind, the present study aims to (i) identify potato genotypes with high P uptake efficiency within a diversity set that includes cultivated potatoes and their wild relatives, (ii) study the genetic variation between the genotypes, and (iii) identify markers significantly associated with P deficiency tolerance.

## Materials and methods

### Potato diversity set

The diversity panel used in this study consisted of clones of 183 different potato accessions belonging to *Solanum tuberosum* spp. *tuberosum* L. (*tbr*, 178 genotypes), *S. chacoense* (Bitter) (*chc*, three genotypes), *S. pinnatisectum* Dunal (*pin*, one genotype), and *S. stenotomum* Juz. & Bukasov (*stn*, one genotype) from the Gross Luesewitz Potato Collections (GLKS) of the Leibniz Institute of Plant Genetics and Crop Plant Research (IPK), Germany (Table S1). The cultivated accessions (*Solanum tuberosum* spp. *tuberosum*) will be denoted as KKS and Andean landraces/the wild relatives as AKS/WKS. The genotypes were compared to 17 modern starch potato cultivars from breeders. The genotypes originated from diverse geographical regions, with 163 coming from Europe, 25 from the Americas, 10 from Asia, and 2 with unknown origin. Out of all cultivated potato genotypes, 69 are categorized as starch potatoes, 59 as table potatoes, and 24 have multiple applications. Additionally, 12 genotypes are primarily cultivated for industrial processing purposes and 36 genotypes do not possess any known specialty. The potato genotypes belong to varying maturity groups and were registered as variety in different years from 1849 (GLKS 11704) to 2018 (Standard 16) (Table S1).

### Phenotypic screening

#### Experimental design

The study materials were maintained as in vitro cultures in MS medium [38] under controlled climate condition. For phenotypic screening, four-weeks old in vitro plantlets (approx. 15 cm tall) were planted in pots (15 cm x 15 cm x 21 cm) with drainage holes with 3.75 kg of a sand substrate per pot. This was comprised of three different size fractions of sand particles (0.4–0.8 mm; 0.7–1.25 mm; 1.2–2.5 mm) in the ratio 1:1:1. Two plants (one tip and one stem cutting; sum of the two plants per pot was treated as one observational unit) per pot were grown for four weeks. The experiment followed a randomized block design with two consecutive biological replicates from March to June 2020 in a greenhouse with 21 °C and additional light when natural light was below 5 klux for max. 12 h. For the fertilizer treatment, a modified Hoagland solution [39] was applied. The accessions were grown under two P treatments, i.e. high P (HP, 100%) with 15 mg P as KH_2_PO_4_ L^−1^ and low P (LP, 20%) with 3 mg P as KH_2_PO_4_ L^−1^ in the nutrient solution. Both treatments were supplemented with 209.94 mg N as KNO_3_, 214.83 mg K as K_2_SO_4_, 48.36 mg Mg as MgSO_4_7H_2_O, 64.45 mg S as ZnSO_4_7H_2_0, 200.26 mg Ca as Ca(NO_3_)_2_4H_2_O per L of the nutrient solution. Additionally, micronutrients were added to both treatments in the following amounts per L of nutrient solution: 0.500 mg B as H_3_BO_3_, 0.502 mg Mn as MnSO_4_2H_2_O, 0.050 mg Zn as ZnSO_4_7H_2_O, 0.012 mg Cu as CuSO_4_5H_2_O and 0.012 mg Mo along with 0.013 mg Na as Na_2_MoO_4_2H_2_0. The pH of the nutrient solutions was maintained at 5.8. The plants were irrigated with 100 ml of the nutrient solution every second day. The nutrient solution could freely drain through the drainage holes. Altogether, with 200 potato genotypes, 2 in vitro plantlets per genotype and pot, two P treatments and two consecutive replicates, the phenotyping relies on 1600 plants. The experimental design described above also enabled us to schedule the experimental period at a time of year (spring to early summer) when air temperatures and the duration and intensity of lighting in the greenhouse could be kept at a comparable level. In addition, relatively large and tall pots were chosen in order to obtain a well-developed root system that is capable of exploring a large substrate volume.

#### Phenotypic evaluation

During and before harvesting, various above and underground parameters were measured (Table 1). Plant height was measured twice, i.e. 7 and 30 d after planting (PH1 and PH2). After 30 d, the two plants per pot were harvested and pooled as one sample. Shoot biomass, root biomass, and tubers (if produced) were harvested separately. During harvesting, root length (RL) was measured for each pot by using a ruler. The fresh weight (FW) was measured for each sample by weighing. The samples were dried at 60 °C for 3 days and the dry weight (DW) was determined by weighing again afterwards.

**Table 1:** List of measured traits, their units and description

Trait	Abbreviation	Unit	Description
Shoot fresh weight	SFW	g	Fresh weight of the harvested shoot biomass
Root fresh weight	RFW	g	Fresh weight of the harvested root biomass (after washing to remove the sand particles and dry patting)
Shoot dry weight	SDW	g	Weight of the dried shoot biomass (60^o^C)
Root dry weight	RDW	g	Weight of the dried root biomass (60^o^C)
Root-to-shoot ratio	R: S	-	Root dry weight divided by shoot dry weight
Tuber fresh weight	TFW	g	Fresh weight of the harvested tubers
Tuber dry weight	TDW	g	Weight of the dried tubers (60^o^C)
Total fresh weight	TotalFW	g	Total weight of the fresh plant biomass
Total dry weight	TotalDW	g	Total weight of the dried plant biomass
Plant height at 7 days after planting	PH1	cm	Average height of the two plants in the pot (stem and tip cutting) measured up to the newest leaf
Plant height at 30 days after planting	PH2	cm	Average height of the two plants in the pot (stem and tip cutting) measured up to the newest leaf
Root length	RL	cm	Length of the longest root from the base of the shoot
Phosphorus concentration	P_conc	mg (100 g) ^−1^	Amount of phosphorus present in shoot biomass
Phosphorus uptake	Pupt	mg plant^−1^	Amount of phosphorus taken up by the plant
Phosphorus utilization efficiency	PUE	g mg^−1^	Amount of biomass produced per unit of phosphorus taken up

Shoot P concentration was measured at the University of Rostock (Germany). The whole dried shoot samples were crushed into a porcelain crucible, dried again at 105 °C, weighed and incinerated at 550 °C for 4–5 h in a muffle furnace. Total P was extracted in 25% hydrochloric acid according to Page et al. [40]. Phosphorus concentrations were measured using inductively coupled plasma-atomic emission spectroscopy (ICP OES Optima 8300, Perkin Elmer) at 214 nm wavelength. P uptake was calculated by multiplying the P concentration of the shoots with its respective dry matter content. Phosphorus utilization efficiency (PUE; g mg^−1^) was calculated as dry shoot weight (g) over P uptake (mg plant^−1^) and indicates how much biomass was produced per mg P taken up. For the traits shoot and root biomass, total biomass and P uptake, Stress Tolerance Indices (STI) according to Fernandez [41] were calculated as:

$$\mathrm{STI}\;=\:\frac{Y_{pi\:\ast}{\:Y}_{si}}{{Mean\:Y_p}^2}$$

where Y_pi_ is the performance of the i^th^ population, i.e. the genotype i under control condition (HP); Y_si_ is the performance of the i^th^ population under P deficiency (LP) and Mean Y_p_ is the mean performance of all the genotypes in the control condition. Genotypes with high STI values were considered to have superior performance under HP and LP conditions. Based on the STI for the traits shoot and root biomass, total biomass and P uptake, the membership function value for P stress tolerance (MFVP) was calculated according to Chen et al. [42] as:

$$\:\text{U}\text{i}\text{j}\:=\:\frac{{STI}_{ij}-{STI}_{jmin}}{{STI}_{jmax}-{STI}_{jmin}}\:,\:{U}_{i}=\frac{1}{n}\sum\:_{j=1}^{n}{U}_{ij}$$

where U_ij_ is the membership function value of the trait (j) for the genotype (i) for P stress tolerance; STI_ij_ is the stress tolerance index of the trait (j) for the genotype (i); STI_jmax_ is the maximum value of the stress tolerance index for the trait (j); STI_jmin_ is the minimum value of STI_ij_; U_i_ is the average value of the membership function of traits for the genotype (i) for P stress tolerance.

MFVP assesses the overall stress tolerance of a genotype, encompassing multiple phenotypic traits.

#### Statistical analysis of phenotypic traits

The data was checked visually for normal distribution of residuals and homogeneity of variances using R (version R-4.2.2) [43] in the RStudio environment. Analysis of variance (ANOVA) was performed using the “aov” function [43] in R to assess significant differences among genotypes (G), treatments (T), replications (E), and genotype treatment interaction (G x T) under each fertilizer conditions (HP and LP), with G and G x T interaction as fixed effects in the model. The variance components and broad sense heritability was calculated for each trait based on the formula [44, 45]:

$$\begin{aligned} {\upsigma^2}_{\text{G}} &=\;\left({\mathrm{MSQ}}_{\mathrm G}-{\mathrm{MSQ}}_{\mathrm{GT}}\right)/\mathrm{TxR}\\ {\upsigma^2}_{\mathrm{GT}} &=\;\left({\mathrm{MSQ}}_{\mathrm{GT}}-{\mathrm{MSQ}}_{\mathrm R}\right)/\mathrm R\\{\upsigma^2}_{\mathrm{Re}} &=\;{\mathrm{MSQ}}_{\mathrm{Re}}\end{aligned}$$

where σ²_G_ = Genotypic variation; σ²_GT_ = Variance component of interaction; σ²_Re_ = Residual variance; MSQ_G_ = Mean sum of squares of genotypes from analysis of variance; MSG_GT_ = Mean sum of squares of interaction between genotype and treatment from analysis of variance; MSQ_Re_ = Mean sum of square of the residual error from analysis of variance; T = number of treatments; R = number of replications of each treatment. Broad sense heritability was calculated for each trait as:

$${\mathrm H^2}_{\mathrm{bs}}\;=\;{\upsigma^2}_{\mathrm G}/{\upsigma^2}_{\mathrm P}$$

where H²_bs_ = broad sense heritability; σ²_P_ (phenotypic variance) = $\left[{\upsigma^2}_{\mathrm G}+{\upsigma^2}_{\mathrm{GT}}/\mathrm T\right]\;+\;\left[{\upsigma^2}_{\mathrm{Re}}/\mathrm{TR}\right]$.

Using the R package “heatmaply” [46], a heatmap was produced based on the standardized genotype means for the traits shoot and root dry weight (SDW, RDW), total dry weight (including tubers, TotalDW), and P uptake under HP and LP conditions of the pot experiment. The package used hierarchical clustering to create a heatmap based on the pairwise Euclidean distances between all the rows and columns, which were standardized based on Z-score normalization.

### Genotypic data and genetic diversity analysis

#### Leaf sampling, DNA extraction and genotyping by sequencing

DNA was extracted from ~ 100 mg of freeze-dried leaf tissue according to [47] using a Janus8Tip pipetting robot (PerkinElmer, Waltham, USA). Agarose gel (1%) electrophoresis was performed using 1x Tris-acetate-EDTA buffer to check the quality of the extracted DNA. The samples were quantified using Hoechst dye in a VICTOR^®^ Nivo™ multimode plate reader (PerkinElmer, Waltham, USA) and diluted to 20 ng µl^−1^, with 30 µl of these solutions being prepared for sequencing.

The panel of the 200 potato genotypes was sequenced at the “Genomics of Genetic Resources” (GGR) Research Group at IPK Gatersleben using a NovaSeq600 (Illumina, San Diego, CA, USA). To obtain genome-wide SNPs, genotyping-by-sequencing (GBS) analyses [48] were conducted for the 200 genotypes. For the library preparation, 200 ng of genomic DNA were used and cut with the two restriction enzymes *Pst* l*-*HF (NEB) and *Msp* I (NEB). Library preparation, individual barcoding, and 100 bp paired-end sequencing was performed following Wendler et al. [49].

#### Data assembly and analysis

The quality of the GBS data from Illumina sequencing was assessed using FASTQC v.0.11.7 [50]. Paired-end raw reads of 200 samples were demultiplexed using the Casava pipeline 1.8 (Illumina), trimmed, and filtered using Cutadapt [51] within Ipyrad version 0.7.28 [52] (http://ipyrad.readthedocs.io/), a toolbox for assembly and analysis of RAD-seq data sets based on the pyRAD pipeline [53]. Data was reference assembled by aligning the sequences to the potato reference genome SolTub_3.0. Filtered reads were clustered at 90% thresholds within each sample. Clusters with a minimum depth of less than five were discarded. Error rate and heterozygosity were jointly estimated based on counts of site patterns across clustered reads for each sampled individual and the average parameter values were used for consensus base calling. Consensus loci were then clustered across samples at 90% similarity and aligned. The dataset was filtered again for loci with the minimum number of 120 samples that must have data at a given locus for it to be retained in the final data set and maximum of 20% SNPs per locus.

#### Population structure analysis

To investigate the genetic stratification of the potato panel, two different approaches were applied, namely Principal Component Analysis (PCA) and a model-based Bayesian population assignment using the R package “LEA” [54]. Population assignment was performed for K = 1 to 10 with 10 repetitions each and ploidy set to four. The optimal K was then determined by the lowest entropy value using the snmf () function, to determine the optimal number of ancestral populations. The Q matrices obtained with LEA (for K = 6, K = 10), which include the ancestral assignment frequencies, were sorted using the R package “tidyverse” [55] and plotted with “ggplot2” [56], discerning different ancestral clusters with color-coding. The “ggplot2” package was also used for plotting the PCA results.

#### Phylogenetic analysis

The SNP data set from the raw GBS data was analyzed with IQ-TREE v2.2.6 [57, 58] to infer relationships between the accessions using a maximum-likelihood (ML) framework. A filtered VCF file, generated by VCFTOOLS [59] was converted to PHYLIP format via the Python script vcf2phylip.py [60] input into IQ-TREE. To identify the optimal model for phylogenetic inference, MODELFINDER [61] was applied, selecting the transversion model with equal base frequencies (TVME) based on the Bayesian information criterion. Clade support was assessed with 1000 bootstrap replicates [62], incorporating a burn-in of 250.

#### Genome wide association studies (GWAS)

The original SNP dataset was filtered with the program PLINK2 in Linux [63] to avoid biased detections due to rare alleles. Markers with a call rate lower than 85% and with a minimum allele frequency (MAF) lower than 5% were discarded. Following filtering for call rate and MAF, and subsequent imputation to reduce missing genotypes per locus, a total of 4,796 SNPs was retained for GWAS analyses. GWAS was performed using “rMVP” package [64] in R. Least-square means were calculated, and after testing the models GLM, MLM and FarmCPU, we used the FarmCPU model for our GWAS analysis due to highest number of significant and reliable MTAs, supported by appropriate Q-Q plot diagnostics [65]. Two separate association analyses were performed for the two different phosphorus treatments (HP and LP). Additional GWAS was performed using trait differences between HP and LP and their stress tolerance indices. The significance threshold to declare a marker as associated was set to 0.05 according to Bonferroni threshold, calculated as -log_10_(0.05/4,796). Based on prior studies, the genes within a window of 1 million bp upstream and downstream of the significant SNPs were screened to search for candidate genes underlying each trait using the database phytozome [66].

#### Estimation of phenotypic variance explained (PVE) by significant associations

The proportion of variance in the particular phenotype explained by each SNP was calculated using various components of the GWAS results according to Shim et al. [67] based on the following equation:

$$\mathrm{PVE}\;\left(\mathrm{SNP}\right)\;=\;\frac{2\;\times\;\upbeta^2\;\times\;\mathrm{MAF}\;\times\;\left(1-\mathrm{MAF}\right)}{2\;\times\mathrm\beta^2\;\times\;\left(1-\mathrm{MAF}\right)\;+\;\left(\mathrm{SE}\left(\upbeta\right)\right)^2\times\;2\;\times\;\mathrm N\;\times\mathrm{MAF}\;\times\left(1-\mathrm{MAF}\right)}$$

Where N is the sample size of the panel, β is the effect of the genetic variant (SNP) of interest, SE(ß) is the standard error of the effect of the genetic variant (SNP) of interest, MAF is the minor allele frequency for the genetic variant (SNP) of interest.

## Results

### Phenotypic screening of 200 potato accessions

**Fig. 1:**
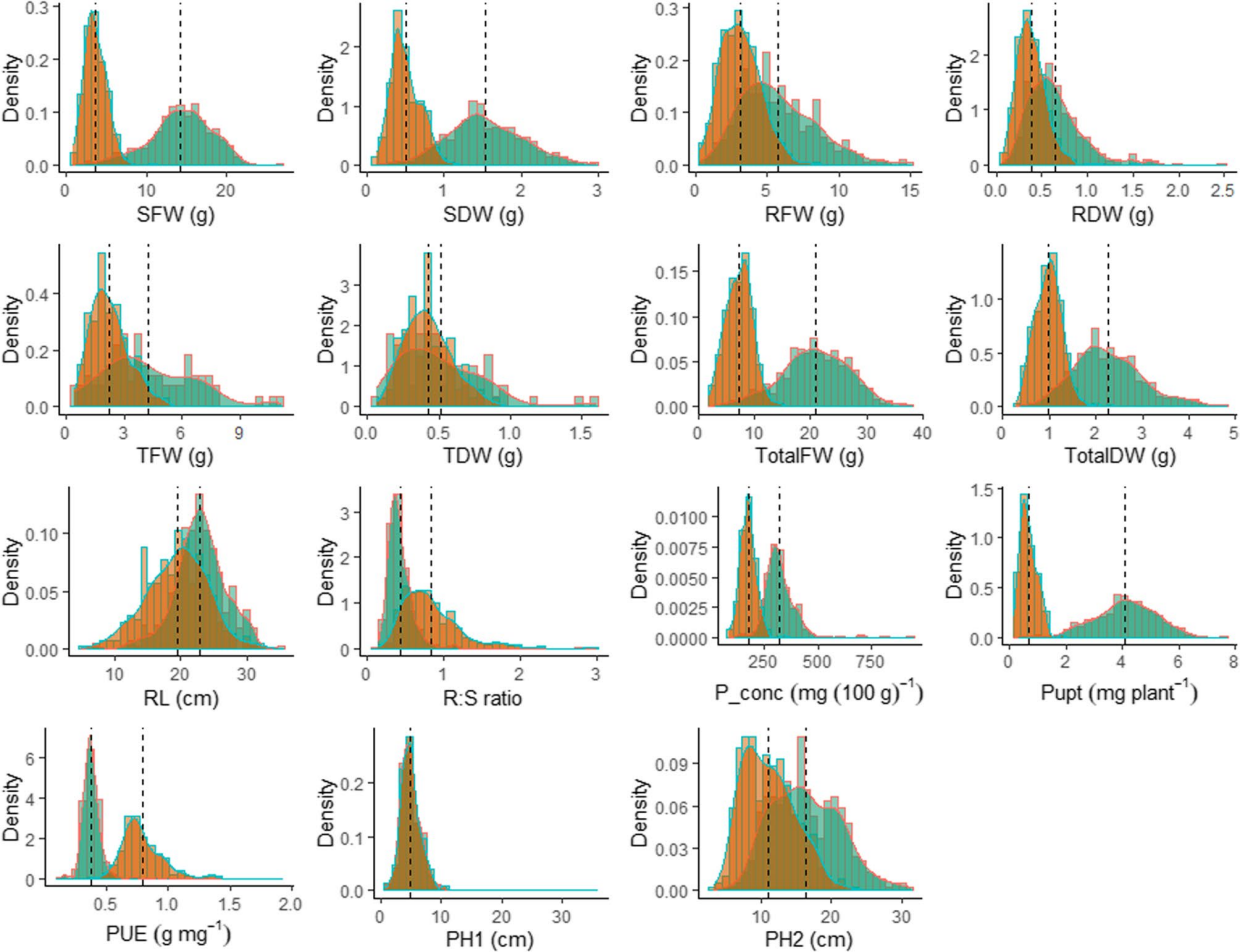
Normal distributions fitting the data for the traits: shoot weight (SFW), shoot dry weight (SDW), root fresh weight (RFW), root dry weight (RDW), tuber fresh weight (TFW), tuber dry weight (TDW), total fresh weight (TotalFW), total dry weight (TotalDW), root length (RL), root-to-shoot ratio (R: S ratio), phosphorus concentration (P_conc), phosphorus uptake (Pupt), phosphorus utilization efficiency (PUE), plant height after 1 week (PH1), plant height after 4 weeks (PH2) under HP (green) and LP (orange) conditions. Dashed lines represent the mean values of the traits

The shoot dry weight varied across genotypes, ranging from 2.44 g per pot to 0.42 g per pot (Table 2, Table S1) under HP conditions for GLKS 38,155 (*S. chacoense*) and GLKS 11,578 (Kristall). Shoot dry weight decreased under LP conditions and ranged from 0.86 g per pot to 0.22 g per pot (Table 2, Table S1) for GLKS 38,155 (*S. chacoense*) and GLKS 11,015 (Kresnik). Root dry weight under HP was highest for of the genotype GLKS 10,800 (Limba) with 1.83 g per pot, and lowest for GLKS 11,015 (Kresnik) with 0.17 g per pot (Table 2, Table S1). Root dry weight generally decreased under LP conditions and ranged from 0.93 g per pot for GLKS 10,800 (Limba) to 0.12 g per pot for GLKS 11,558 (Kero) (Table 2, Table S1). The results showed a reduction in shoot and root dry weight by 66% and 36% under LP conditions on average, respectively (Table S1). Interestingly, ten genotypes exhibited a higher root dry weight under LP conditions compared to HP conditions, namely GLKS 11,704 (Blanchard), GLKS 11,723 (Paterson’s Victoria), GLKS 11,918 (Tylva), GLKS 11,422 (Erasme), GLKS 11,495 (Hokkaiaka), GLKS 11,603 (Liwia), GLKS 11,634 (Marfona), GLKS 11,928 (Ulster Torch), GLKS 24,129 (*S. stenotomum*), and GLKS 10,603 (Paul Krüger) (Table S1). Most genotypes exhibited a higher root-to-shoot ratio under stressed conditions, with an average increase of approx. 47% under LP conditions (Table S1). ANOVA results showed significant differences in shoot dry weight among the genotypes under both LP and HP conditions (Table 2). The RDW showed significant differences among the genotypes under HP and LP conditions, however, all the genotypes responded in a similar pattern to the treatments (Table 2). The PH at harvest (PH2) were clearly higher for most genotypes under HP conditions compared to LP conditions, with significant differences observed among the genotypes (Table 2; Fig. 1). These ranged from 26.63 cm (GLKS 10873, Fransen) to 7.00 cm (GLKS 11569, Komsomolets20) under HP conditions and 18.75 cm (GLKS 10873, Fransen) to 3.75 cm (GLKS 11015, Kresnik) under LP conditions (Table 2, Table S1). Total root length of 155 genotypes decreased under LP conditions. However, 31 genotypes showed an increase in root length under stress conditions along with decrease in their shoot biomass which ranged from 36.10% (GLKS 11704, Blanchard) to 76.43% (GLKS 11982, Zarewo) (Table S1).

**Table 2:** Descriptive statistics, ANOVA and broad sense heritability (H^2^_bs_) of the phenotypic traits for genotype (G), treatment (T), replication (E) and G x T interaction effect

Trait^a^	HP				LP				ANOVA^b^				
	Min	Mean	Max	SD	Min	Mean	Max	SD	G	T	E	G x T	H_bs_^2^
SFW [g]	2.48	14.28	23.39	3.34	1.02	3.66	8.11	1.08	***	***	***	***	0.61
SDW [g]	0.42	1.53	2.44	0.38	0.22	0.50	0.86	0.12	***	***	***	*	0.55
RFW [g]	1.58	5.76	12.91	2.12	0.54	3.14	6.54	1.04	***	***	NS	NS	0.69
RDW [g]	0.17	0.64	1.83	0.25	0.12	0.38	0.93	0.12	***	***	***	NS	0.71
Root: Shoot	0.16	0.42	1.71	0.15	0.40	0.83	2.00	0.25	**	***	***	NS	0.58
TFW [g]	0.76	4.41	11.14	2.16	0.99	2.25	5.01	0.87	*	***	***	NS	0.58
TDW [g]	0.07	0.52	1.60	0.30	0.13	0.43	0.88	0.15	***	***	***	**NS**	0.51
TotalFW [g]	5.63	20.81	33.71	5.19	2.46	7.30	11.96	1.78	***	***	NS	**	0.60
TotalDW [g]	0.92	2.26	3.97	0.60	0.41	0.98	1.71	0.22	***	***	***	NS	0.61
RL [cm]	7.00	22.97	29.00	2.97	9.00	19.53	31.00	3.75	***	***	NS	NS	0.63
P_conc [mg 100 g^−1^]	205.46	321.04	543.64	51.59	115.03	172.43	286.63	28.34	**	***	***	NS	0.64
Pupt [mg plant-^1^]	0.71	4.10	5.77	0.83	0.19	0.67	1.34	0.19	***	***	***	**	0.44
PUE [g mg^−1^]	0.27	0.37	0.59	0.05	0.43	0.77	1.25	0.13	**	***	NS	NS	0.33
PH1 [cm]	2.38	4.98	18.95	1.66	1.98	4.93	9.00	1.37	***	NS	***	NS	0.77
PH2 [cm]	7.00	16.35	26.63	3.36	3.75	11.08	18.75	2.69	***	***	***	NS	0.88

^a^Trait abbreviations are explained in Table 1

^b^***p < 0.001, **p < 0.01, *p < 0.05, *NS* Not significant

The frequency distribution for most of the traits under HP condition exhibited approximately normal distribution, with a higher mean and wider range compared to the LP condition (shoot biomass, root biomass, phosphorus uptake; Fig. 1). The total biomass and the tuber biomass show a slightly positively skewed distribution (Fig. 1). The root length under control conditions exhibited a slightly negatively skewed distribution compared to the relatively normal distribution of the root lengths under stressed condition (Fig. 1). This suggests a complex genetic control and a combination of different factors influencing the observed phenotypic variation in the traits (Fig. 1).

**Fig. 2:**
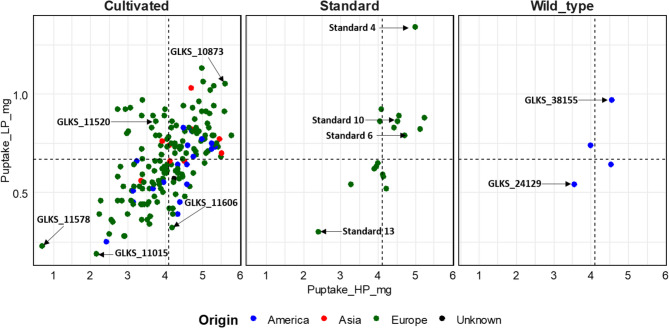
P uptake of 200 different potato genotypes under HP (15 mg/l P as KH_2_PO_4_) and LP (3 mg/l P as KH_2_PO_4_) conditions, cultivated under greenhouse conditions

The P uptake among the genotypes was significantly higher under HP than under LP conditions (Fig. 1; Table 2). It varied from 5.77 to 0.71 mg per plant for GLKS 11,607 (Lorch) and GLKS 11,578 (Kristall) under HP conditions, and between 1.34 and 0.19 mg per plant under LP conditions for Standard 4 and GLKS 11,015 (Kresnik), respectively (Table 2, Table S1). A number of genotypes with high P uptake under both HP and LP conditions was identified from the dotplot for P uptake under HP and LP (Fig. 2), namely GLKS 38,155 (*S. chacoense*), Standard 4 and GLKS 10,873 (Fransen). On the contrary, genotypes like GLKS 11,578 (Kristall) and GLKS 11,015 (Kresnik) were characterised by low P uptake under both stress and control conditions (Fig. 2).

**Fig. 3:**
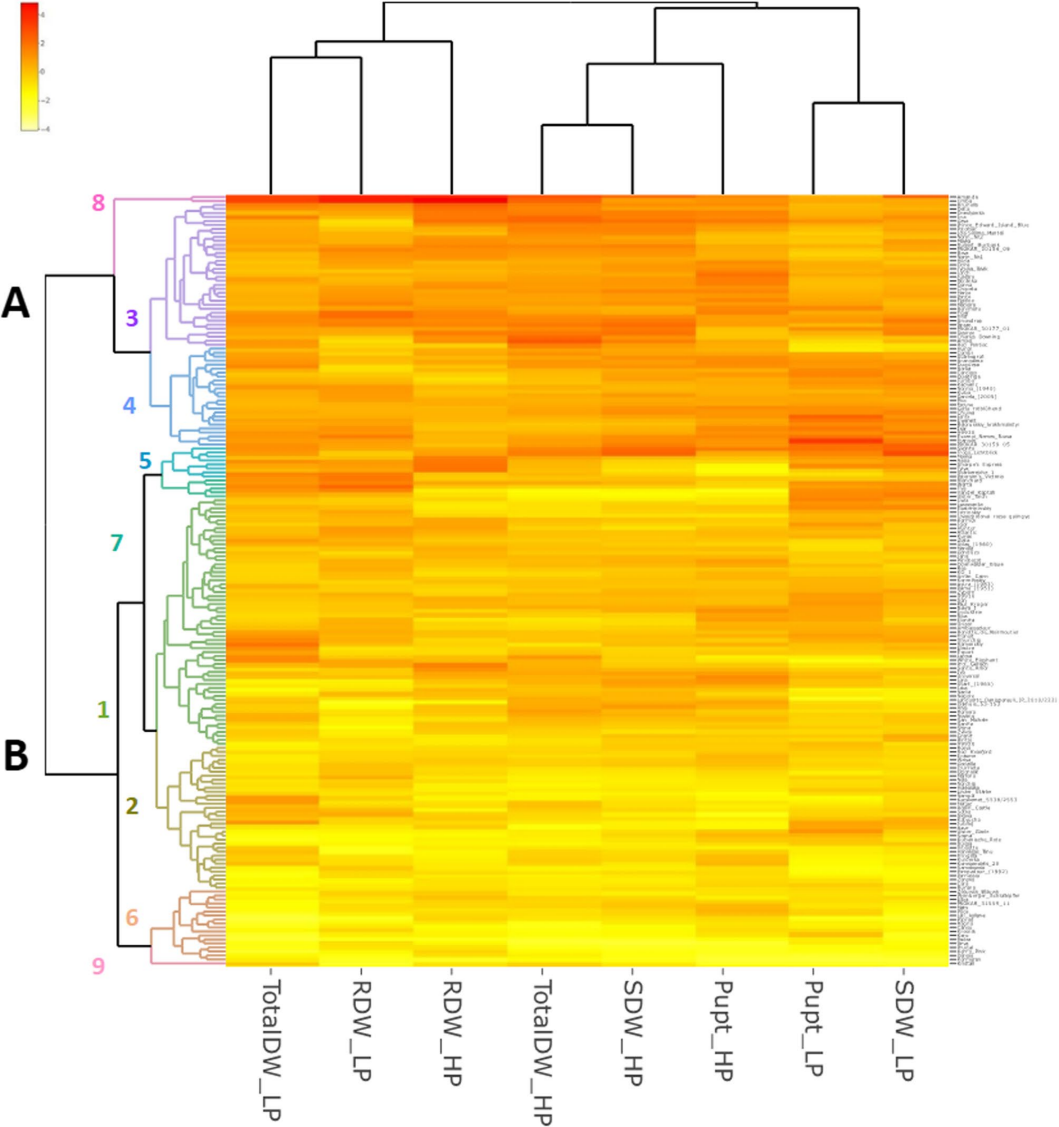
Heatmap based on standardised genotype means of 200 potatoes for the traits shoot and root dry weight (SDW, RDW), total dry weight including tubers (TotalDW) and P uptake (Pupt) in the phenotyping experiment under HP and LP conditions; bright red indicates higher values and yellow indicates lower values

Using hierarchical clustering of the standardised trait values for P uptake, shoot and root dry weight and total dry weight, classified the accessions into two main clusters and 9 sub-clusters (Fig. 3, Table S1). Main cluster A comprised 63 genotypes and cluster B had 130 genotypes. Sub-clusters 3, 4 and 8 (Cluster A), sub-clusters 1 (Cluster B), 5 and 7 (Cluster A) and sub-2, 6 and 9 (Cluster B) were identified as the best, intermediate and the worst performing clusters, respectively, based on the mean values of the traits for each sub-cluster (Table 3; Fig. 3). Genotypes from sub-cluster 3 demonstrated high P uptake along with high biomass under HP, suggesting strong P acquisition (Table 3). Genotypes from sub-clusters 3 and 8 like GLKS 11,242 (Amanda), GLKS 10,800 (Limba) and GLKS 10,591 (Tiger) were characterised by high root dry weight compared to that of the genotypes from the other clusters. Most of the Andean/wild accessions were part of cluster A, except GLKS 24,129 (*S. stenotomum*), which belonged to sub-cluster 6 (Fig. 3, Table S1). Nine genotypes of modern varieties fell into the intermediate sub-clusters, while seven genotypes belonged to the best performing sub-clusters and four genotypes to the worst performing sub-clusters (Fig. 3, Table S1).

**Table 3:** Mean values of shoot dry weight (SDW), root dry weight (RDW), total dry weight (TDW) and phosphorus uptake (Pupt) under HP and LP across heatmap clusters (1–9), with highest values highlighted in bold

Cluster	Mean SDW_HP (g)	Mean SDW_LP (g)	Mean RDW_HP (g)	Mean RDW_LP (g)	Mean Pupt_HP (mg plant^−1^)	Mean Pupt_LP (mg plant^−1^)
1	1.517	0.478	0.608	0.361	4.179	0.650
2	1.218	0.412	0.432	0.295	3.556	0.544
3	**1.990**	0.563	**0.907**	0.467	**4.874**	0.719
4	**1.883**	**0.653**	0.688	0.442	**4.874**	**0.929**
5	1.434	0.590	**0.873**	**0.541**	3.491	0.756
6	0.948	0.310	0.380	0.252	2.837	0.383
7	1.170	**0.649**	0.426	0.417	3.309	**0.924**
8	**2.123**	**0.680**	**1.723**	**0.915**	**5.178**	**0.765**
9	0.415	0.265	0.670	**0.500**	0.705	0.230

### Evaluation of P stress tolerance in the set of 200 potato genotypes/membership function value for P stress tolerance (MFVP)

The stress tolerance index (STI) provides information on how tolerant a respective genotype is to P shortage in relation to the mean performance of all tested genotypes regarding a respective trait. Based on the stress tolerance index for P uptake, the modern cultivar Standard 4 and GLKS 10,873 (Fransen) were identified as the most tolerant ones with STI values of 0.40 and 0.35 (Table S1). The most susceptible genotypes GLKS 11,578 (Kristall) and GLKS 11,015 (Kresnik) had STI values for P uptake of 0.01 and 0.02 respectively (Table S1).

**Fig. 4:**
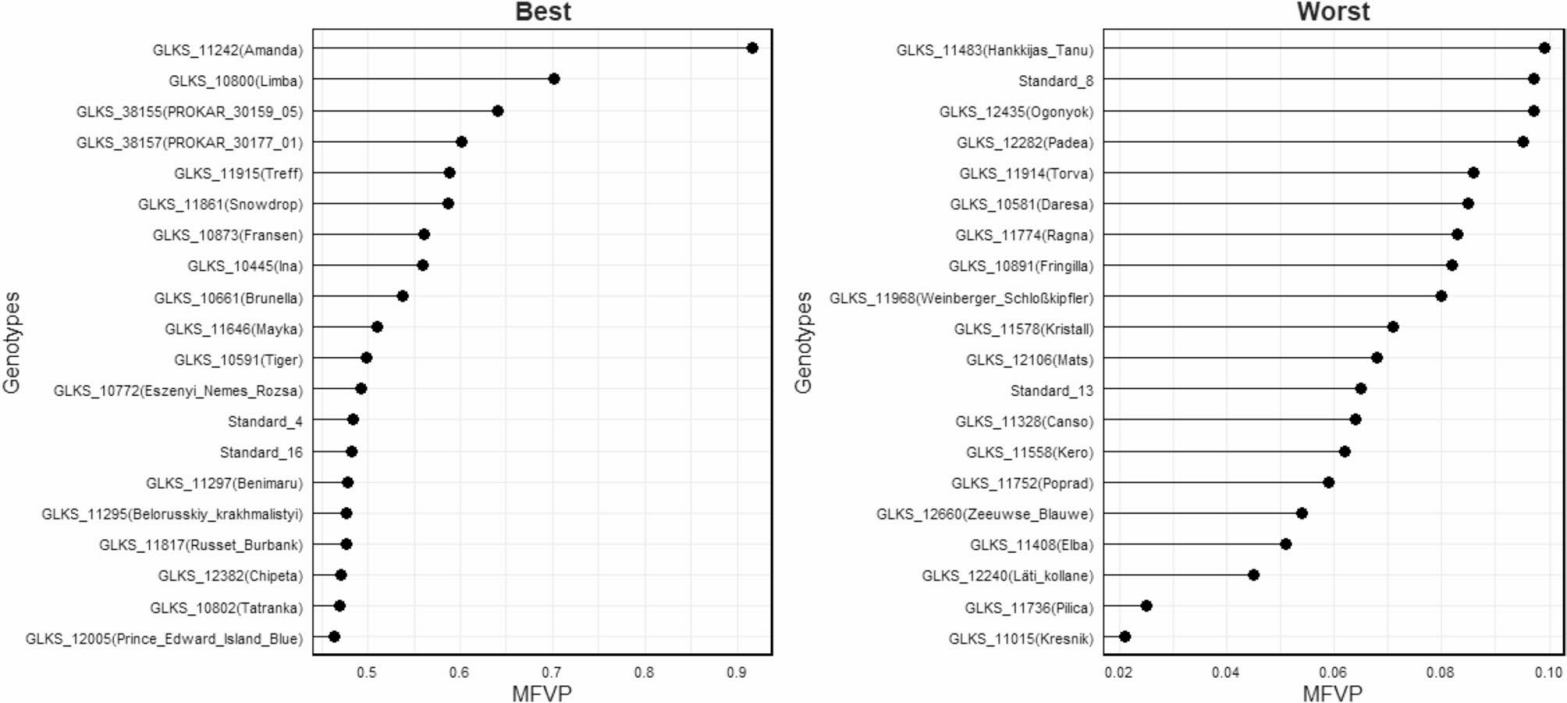
Membership function value (MFVP) for low P tolerance according to Chen et al. [42] based on the differences in shoot and root dry weight, total dry weight and P uptake of potato genotypes under high and low P conditions. Shown are the genotypes with 20 highest and 20 lowest MFVP values (Table S1)

The MFVP ranged between 0.02 and 0.92 for GLKS 11,015 (Kresnik) and GLKS 11,242 (Amanda) (Table S1). Based on the theory that MFVP can be used as a comprehensive index to evaluate the low P tolerance for the potato genotypes [42], 20 genotypes with highest MFVP were identified as the most tolerant genotypes under phosphorus stress conditions. Similarly, 20 genotypes with lowest MFVP were identified as least low P tolerant genotypes (Fig. 4, Table S1).

### Genotypic data and genetic analysis

An ipyrad analysis with 199 potato GBS sequences with a threshold of 120 samples sharing a locus was carried out, which resulted in a dataset comprising 8956 loci with 21.67% missing sites, an alignment of concatenated loci of 200,56741 bp and 56,738 SNPs. Another additional ipyrad analysis with 194 sequences, excluding the wild potato accessions was run, the output contains an alignment of concatenated loci of 195,43751 bp with 22.04% missing sites and 43,747 SNPs. 

#### Analysis of genetic population structure and ancestral populations

##### Principal component analysis (PCA)

**Fig. 5:**
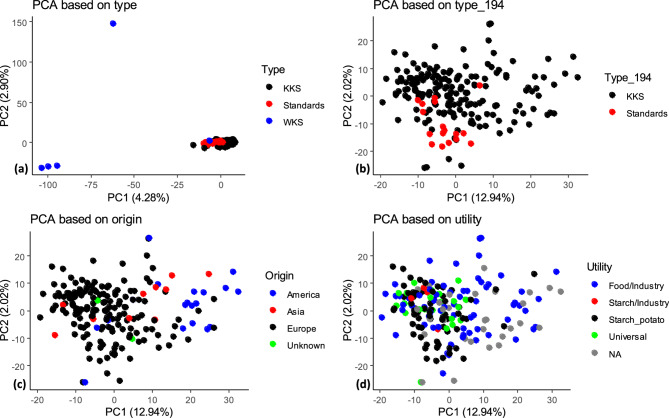
**a** PCA plot depicting distinct clusters including all used potato accessions: cultivated (KKS), native/wild potatoes (AKS/WKS) and standard varieties, **b** PCA plot including only cultivated (KKS) and standard varieties, **c** PCA plot showing geographic origin of the genotypes and excluding AKS/WKS entries **d** PCA showing utilisation type of the genotypes, AKS/WKS entries are excluded

PCA of all 199 analysed accessions revealed three genetic clusters (Fig. 5a). Cultivated accessions (KKS) and the modern varieties formed one large cluster. Among the wild genotypes, all *S. chacoense* entries, i.e. GLKS 38,153, GLKS 38,155 and GLKS 38,157 clustered together in the lower left corner. GLKS 38,157 (*S. pinnatisectum*) is separated in the upper left corner. Only GLKS 24,129, which belongs to the *S. stenotomum* species, was found to cluster with cultivated GLKS accessions and modern starch varieties. The percentage of variance explained by PC1 and PC2 was 4.28% and 2.90%, respectively. In order to gain better insight into the grouping pattern within the cultivated genebank accessions and modern varieties, another PCA was created without the native and wild accessions (PC1: 12.94% and PC2: 2.02%) (Fig. 5b). The PCA plot revealed now relatively weak clusters among the cultivated genebank accessions. The same PCA - showing the origin of the accessions - revealed a moderately good separation of the American varieties from those originating from Europe. Accessions originating from Asia did not form a separate cluster (Fig. 5c). PCA displaying the utilisation of the accessions revealed some weak but not clear distinction between groups. However, the starch potato varieties tended to cluster together with the universal utility varieties (Fig. 5d).

##### Population ancestry

**Fig. 6:**
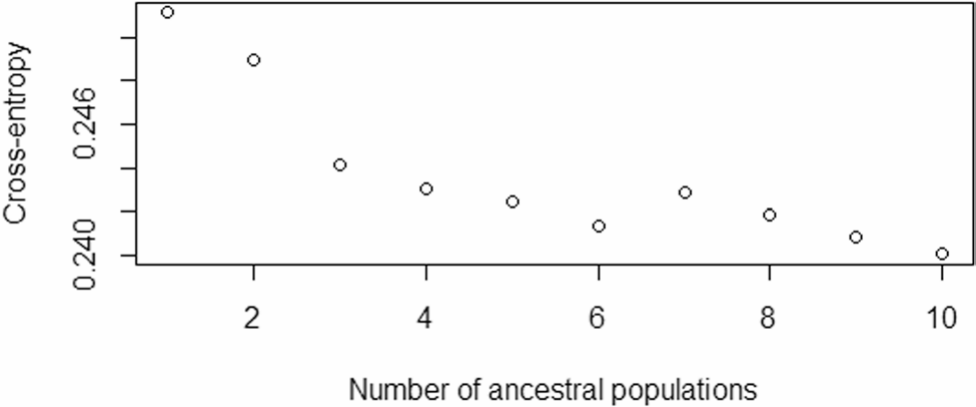
Cross-entropies as a function of the number of populations in snmf

The numbers of ancestral populations that best explain the genotypic data were chosen to be 10 and 6, as indicated in Fig. 6 [68, 69].

**Fig. 7:**
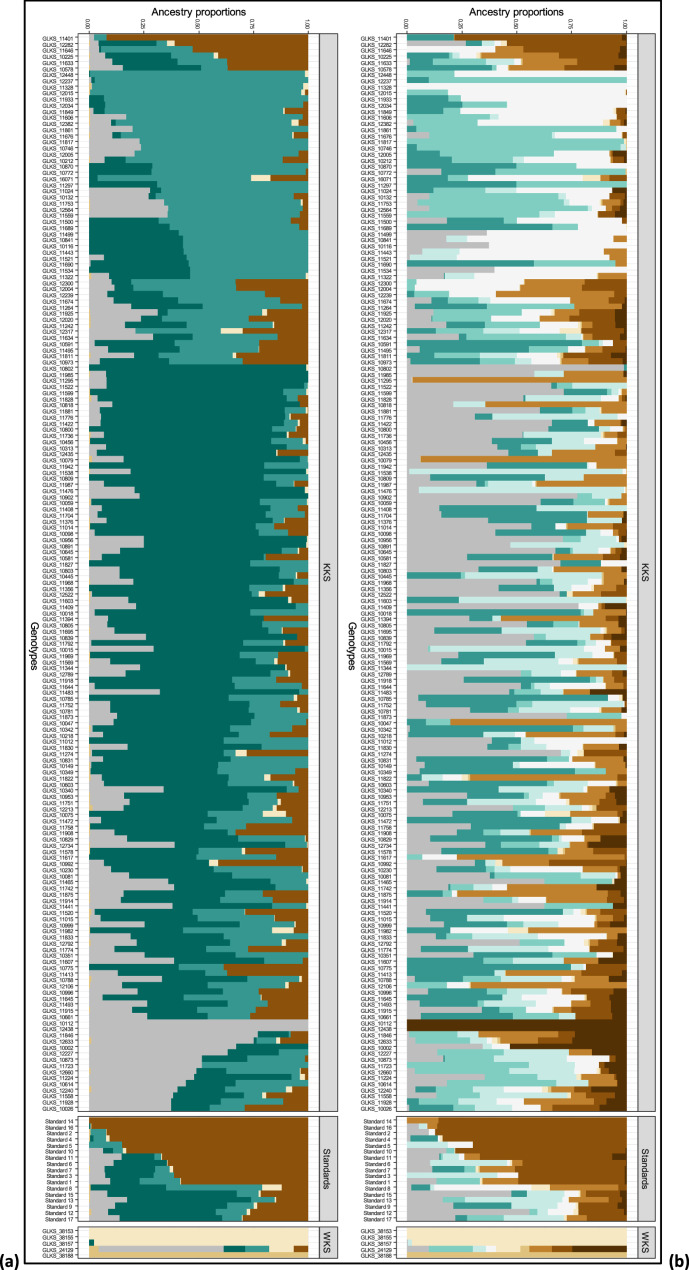
Ancestry coefficients of 199 potato accessions consisting of cultivated accessions (KKS), Andean/wild accessions (AKS/WKS), and modern starch varieties (standards), obtained from snmf (); optimal number of ancestral populations K = 6 (**a**) and K = 10 (**b**) respectively

Ancestral population assignment (Fig. 7), identified distinct ancestral affiliations among the cultivated gene bank accessions, wild relatives and modern accessions indicating high genetic diversity. Among the cultivated types, seven major ancestral clusters were recorded (for K = 10). The modern starch varieties (standards) were admixed or mainly assigned to one major cluster (Fig. 7a (brown color) and Fig. 7b (brown color)). Three of the wild accessions were assigned to ancestral populations that were not present in any other sample. One ancestral population includes the three genotypes belonging to *S. chacoense*: GLKS 38,153, GLKS 38,155 and GLKS 38,157. The other accession that does not have any common ancestry with other samples (GLKS 38188) belongs to *S. pinnatisectum*. In contrast, *S. stenotomum* GLKS 24,129 shares a similar ancestry with the cultivated accessions. Considering K = 6, the barplot (Fig. 7a) indicated four distinct ancestral populations contributing to the genepools of the cultivated accessions. Some of the modern cultivars shared ancestry with the cultivated gene bank accessions of the present study, while few of them were assigned to one major ancestral cluster, which was shared by a few cultivated ones. Regarding the wild accessions, the pattern of ancestry was found to be similar to K = 10 (Fig. 7b).

#### Phylogenetic analysis

**Fig. 8:**
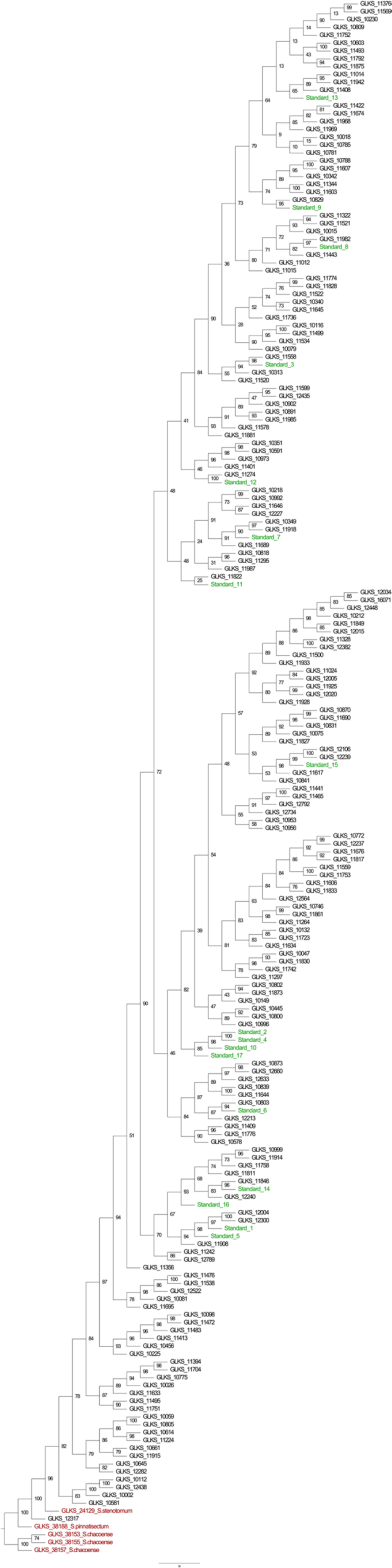
Phylogenetic tree of 199 potato accessions; black, red and green colors represent cultivated accessions (KKS), Andean/wild accessions (AKS/WKS) and modern starch varieties (standards), respectively

Based on the phylogenetic tree, a high level of branching can be observed, which indicates extensive diversification and evolutionary divergence among the genotypes (Fig. 8). The wild accessions belonging to *S. chacoense* (GLKS 38153, GLKS 38155, GLKS 38157) formed a strong supported clade (Fig. 8). The *S. pinnatesectum* (GLKS 38188) was found in a separated clade which supports the findings of the ancestral population assignment where it didn’t share ancestry with any other accession (Figs. 7 and 8). The *S. stenotomum* (GLKS 24129) was found be in a sister clade that closely related to the cultivated entries thus, confirming ancestral population assignment (Fig. 7) and the PCA (Fig. 5a).

#### Identification of Marker-Traits associations (MTAs) related to phosphorus stress

Genome-wide association studies using the FarmCPU model identified 27 unique significant MTAs (above the experimental threshold; Fig. S1), with model fitness for each trait illustrated by the quantile-quantile plots in Fig. S2. Two SNPs were significant for multiple traits, 15 of the MTAs were related to stress tolerance indices of the phenotypic traits (Table 4). Most of the associations were found to be related to shoot traits (12 SNPs) and phosphorus utilisation efficiency (7 SNPs). Many genes that were in the same regions as the MTAs were found to be associated with abiotic and biotic stress tolerance (Table 5). The percentage of phenotypic variance explained (PVE) by the SNPs ranged between 11.12% and 6.87% (Table 4).

**Table 4:** Marker trait associations (MTAs) related to various phenotypic traits under HP and LP conditions, their differences (∆) under both conditions and their stress tolerance indices (STI) the chromosome in which they are present (CHROM), reference allele at the SNP position (REF), alternate allele at the SNP position (ALT), standard error (SE), their minor allele frequency (MAF) and the percent phenotypic variance explained by the SNPs (PVE)

Trait	SNP	CHROM	REF	ALT	SE	MAF	PVE (%)
SDW_HP	loc3759_pos83	4	T	A	0.070	0.063	11.122
	loc3759_pos94	4	T	C	0.071	0.060	10.747
PUE_HP	loc971_pos25	1	A	G	0.008	0.126	10.612
	loc971_pos34	1	C	T	0.008	0.126	10.612
P_conc_HP	loc7748_pos10	10	A	T	6.713	0.233	10.560
	loc7748_pos78	10	G	A	6.732	0.236	9.932
	loc4846_pos21	6	A	G	8.074	0.113	9.797
	loc4846_pos45	6	A	G	8.074	0.113	9.797
PUE_HP	loc6966_pos69	9	G	A	0.008	0.160	10.518
	loc1486_pos47	2	T	A	0.010	0.068	10.257
	loc1307_pos91	2	A	G	0.007	0.107	9.733
PH1_LP	loc1659_pos21	2	A	T	0.192	0.215	9.896
PUE_LP	loc642_pos101	1	C	T	0.016	0.264	9.814
∆RFW	loc1058_pos51	1	G	A	0.200	0.147	12.283
∆PUE	loc642_pos101	1	C	T	0.015	0.264	10.324
PUE_STI	loc1290_pos62	2	C	A	0.114	0.068	11.013
PH1_STI	loc8139_pos64	11	A	G	0.108	0.071	10.985
RFW_STI	loc2624_pos27	3	G	A	0.033	0.416	10.389
PH2_STI	loc6767_pos11	9	G	T	0.047	0.081	10.351
TotalFW_STI	loc5675_pos10	7	A	G	0.014	0.474	10.062
	loc5675_pos13	7	G	C	0.014	0.474	10.062
SDW_STI	loc8926_pos11	12	A	C	0.015	0.152	8.199
	loc5675_pos10	7	A	G	0.010	0.474	8.524
	loc8514_pos92	12	G	T	0.011	0.327	9.595
	loc3882_pos47	5	A	T	0.009	0.490	9.438
SFW_STI	loc4509_pos3	6	C	T	0.024	0.058	8.902
	loc3496_pos6	4	T	A	0.014	0.196	8.375
	loc5675_pos10	7	A	G	0.008	0.474	8.247
	loc8786_pos86	12	C	G	0.008	0.453	8.198
	loc2662_pos37	3	C	T	0.008	0.387	6.869

**Table 5:** List of putative candidate genes and their literature review

Trait	SNP	Candidate gene	Description	Literature
PUE_LP	loc642_pos101	*Soltu.DM.01G031350*	Malate dehydrogenase (MDH1)	The mitochondrial malate dehydrogenase 1 gene GhmMDH1 is involved in plant and root growth under phosphorus deficiency conditions in cotton [70]
		*Soltu.DM.01G031740*	Pectinesterase/Pectin methylesterase	High-Density Genetic Mapping Identifies New Major Loci for Tolerance to Low-Phosphorus Stress in Soybean [71]
		*Soltu.DM.01G032430*	F-BOX AND WD40 DOMAIN PROTEIN	Identification, evolutionary profiling, and expression analysis of F-box superfamily genes under phosphate deficiency in tomato [72] Identification of an F-Box Protein that Negatively Regulates Pi Starvation Responses [73]
		*Soltu.DM.01G031190*	Myb-like DNA-binding domain//DUO POLLEN 1	Genome-Wide Association Analysis for Phosphorus Use Efficiency Traits in Mungbean (*Vigna radiata* L. *Wilczek*) Using Genotyping by Sequencing Approach [74] Identification of an F-Box Protein that Negatively Regulates Pi Starvation Responses [73]
PH1_LP	loc1659_pos21	*Soltu.DM.02G018930*	Universal stress protein family (Usp)	Comparative proteome analysis of metabolic changes by low phosphorus stress in two *Brassica napus* genotypes [75]
		*Soltu.DM.02G019930*	Cytochrome P450 like protein	Gene expression profiles in rice roots under low phosphorus stress [76]
		*Soltu.DM.02G020210*	Ankyrin repeat family protein	Transcriptomic Responses to Thermal Stress and Varied Phosphorus Conditions in *Fugacium kawagutii* [77]
		*Soltu.DM.02G020430*	WRKY DANN binding domain (WRKY)	Role of WRKY Transcription Factors in Regulation of Abiotic Stress Responses in Cotton [78]
		*Soltu.DM.02G020660*	Phosphofructokinase	Phosphorus and carbohydrate metabolism contribute to low phosphorus tolerance in cotton [79]
		*Soltu.DM.02G021000*	Glycerophosphodiester phosphodiesterase/Glycerophosphoryl diester phosphodiesterase	Identification of two glycerophosphodiester phosphodiesterase genes in maize leaf phosphorus remobilization [80]
PUE_HP	loc971_pos25	*Soltu.DM.01G042570*	F-box domain (F-box)	Identification, evolutionary profiling, and expression analysis of F-box superfamily genes under phosphate deficiency in tomato [72]
PUE_HP	loc1486_pos47	*Soltu.DM.02G012540*	Exopolyphosphatase (PRUNE, PPX1)	Phosphate Starvation Responses in Plants and Microbe Mediated Phosphorus Recycling in Soil: A Review [81]; Ddp1 Cooperates with Ppx1 to Counter a Stress Response Initiated by Nonvacuolar Polyphosphate [82]
PUE_HP	loc6966_pos69	*Soltu.DM.09G026670*	MFS transporter, PHS family, inorganic phosphate transporter (PHO84)	Complex Regulation of Plant Phosphate Transporters and the Gap between Molecular Mechanisms and Practical Application: What Is Missing? [83] Molecular mechanisms underlying phosphate sensing, signaling, and adaptation in plants [84]
P_conc_HP	loc4846_pos21	*Soltu.DM.06G022860*	F-box domain (F-box)	Identification, evolutionary profiling, and expression analysis of F-box superfamily genes under phosphate deficiency in tomato [72]
SDW_HP	loc3759_pos83	*Soltu.DM.04G036010*	Fe (3+)-Zn (2+) Purple Acid Phosphatase 12	Genome-Wide Analysis of Purple Acid Phosphatase Genes in *Brassica rapa* and Their Association with Pollen Development and Phosphorus Deprivation Stress [85]
P_conc_HP	loc4846_pos45	*Soltu.DM.06G022930*	Acid phosphatase/Phosphomonoesterase	Root-secreted phosphomonoesterases mobilizing phosphorus from the rhizosphere [86]
				Transcriptome-wide identification and expression profiling of *Pinus massoniana* MYB transcription factors responding to phosphorus deficiency [87]
∆RFW	loc1058_pos51	*Soltu.DM.01G045740*	inorganic pyrophastase	Pyrophosphate and pyrophosphatases in plants, their involvement in stress responses and their possible relationship to secondary metabolism [88]
		*Soltu.DM.01G046060*	Mitogen-activated protein kinase (MAPK) kinase MKK4	Characterization and expression analysis of mitogen-activated protein kinase cascade genes in wheat subjected to phosphorus and nitrogen deprivation, high salinity, and drought [89]
		*Soltu.DM.01G047820*	MYB family trancription factor	Transcription factors and their roles in phosphorus stress tolerance in crop plants [90]
				Expression profile and function characterization of the MYB type transcription factor genes in wheat (*Triticum aestivum* L.) under phosphorus deprivation [91]
RFW_STI	loc2624_pos27	*Soltu.DM.03G024100*	MADS box protein	Expression pattern and function analyses of the MADS transcription factor genes in wheat (*Triticum aestivum* L.) under phosphorus-starvation condition [92]
				Structure and expression of phosphoglucan phosphatase genes of *Like Sex Four1* and *Like Sex Four2* in barley [93]
SFW_STI	loc8786_pos86	*Soltu.DM.12G023690*	WRKY transcription factor 1 (WRKY1)	The WRKY6 Transcription Factor Modulates PHOSPHATE1 Expression in Response to Low Pi Stress in Arabidopsis [94]
SDW_STI	loc8514_pos92	*Soltu.DM.12G010050*	ABC transporter G family member 29	Identification of ABC transporter G subfamily in white lupin and functional characterization of L. albABGC29 in phosphorus use [95]

## Discussion

### Effects of P deficiency in potato genotypes

Phenotypic screening of the 200 potato accessions revealed a substantial phenotypic variation among the genotypes grown under high (HP) and low P (LP). In general, the performance of the genotypes was significantly lower in P-deficient conditions, indicating P stress on the plants. The genotypes produced higher shoot and root biomass under HP conditions, supporting the findings of Schenk (2006) who reported a significant increase of yield and shoot P concentration in potatoes with an increase in P supply. In our experiment, the heights of the plants were also significantly decreased under LP conditions at the end of the experiment i.e. after 4 weeks, even though the plants grew similarly under both conditions for the first week. This suggests that the effect of P stress gradually increased over the weeks and was most prominent at the end of the experiment (Table 2; Fig. 1).

Optimal root growth is essential for P efficiency in potato plants. Previous studies have shown that under P deficient soils, plants tend to increase their root lengths as an adaptive measure for better nutrient uptake under stress conditions [31, 96]. In our study, for the majority of the genotypes the root length decreased under LP conditions, while root-to-shoot ratio increased. This is also evident from studies of Kirchgesser et al. [26] with the same panel of genotypes. An increase in root-to-shoot ratio suggests that the plants put more effort into root growth and development at the expense of the shoots in order to facilitate P uptake indicating their adaptation to LP conditions, supporting various previous findings [31, 97]. These varying morphological responses indicated towards underlying genetic differences among genotypes.

Interestingly, ten potato genotypes in the current study demonstrated a counterintuitive increase in root dry biomass under LP conditions compared to HP. This suggests the presence of genotype-specific adaptive mechanisms that enable more efficient functioning under nutrient stress. Such responses may involve enhanced root proliferation, increased root length density, or greater allocation of biomass to roots - all of which are typical strategies to compensate for low external P availability [31].

Moreover, the root lengths of some genotypes increased under LP conditions in our experiment (Table 2, Table S1), indicating a special ability to explore deeper soil layers. A similar trend in results was shown in potatoes for the varieties Amsel, Paterson’s Victoria and Weinberger Schloßkipler by Wacker-Fester et al. [25], where a significant increase in the specific root length was found under LP treatment.

In addition to root traits, genotypic differences were also evident in phosphorus uptake and utilization efficiency. Our panel also showed significant differences among the genotypes, regarding their total P uptake and utilization efficiency (PUE) under HP and LP conditions (Table 2; Fig. 1). The plants had significantly higher P uptake under HP conditions. The significant genotype x treatment interaction indicated that some genotypes adapt better to low P than others. Specifically genotypes GLKS 10,873 (Fransen) Standards 4, 6, 10 and GLKS 38,155 (*S. chacoense*) were identified as examples with relatively high P uptake under LP (Fig. 2). Inversely to the P uptake, the PUE was significantly lower HP compared to LP conditions. This suggests the better utilization of limited P available by the plants under P-deficient conditions as reported by Rose et al. [98]. A similar trend was also observed in various other crops [99, 100].

The observed variation across genotypes allowed the classification into performance-based clusters under both HP and LP conditions. We were able to identify clusters with high, intermediate and low performing genotypes under both high and low P conditions, based on their biomass and P uptake under HP and LP (Fig. 3). As for a few examples, the genotypes GLKS 11,242 (Amanda) and GLKS 10,800 (Limba) showed very high values for the phenotypic traits under both HP and LP condition (Fig. 3). The genotype GLKS 10,873 (Fransen) showed a high uptake under HP and LP conditions, suggesting tolerance under stressed conditions (Fig. 2). The findings regarding the genotype GLKS 10,873 (Fransen) were consistent to the findings by Wacker-Fester et al. [25], as they also identified this genotype as tolerant to P stress with high P uptake under both HP and LP conditions. Contrastingly, genotypes like GLKS 11,578 (Kristall) and GLKS 10,456 (Prudal) were found to be the worst performing genotypes, based on their lowest values of the phenotypic traits (Fig. 3). Interestingly, the genotype GLKS 11,578 (Kristall) was also found to have a small root system under P deficient conditions in a rhizotron experiment by Kirchgesser et al. [26] with HP and LP conditions. Previous studies by Bachmann-Pfabe and Dehmer [101] showed that wild potato germplasm was superior in terms of nitrogen utilization efficiency and tuber starch content as compared to cultivars. Our present study also revealed that the wild potato accession GLKS 38,155 (*S. chacoense*) has a very high P uptake under both HP and LP conditions as compared to majority of the cultivated genotypes (Fig. 2).

To consolidate these findings, we applied the Membership Function Value of Phosphorus (MFVP) index to assess genotype tolerance under P stress. Previous studies have shown that MFVP, which was calculated based on stress tolerance indices (STI) for a genotype, can be used as a comprehensive index to evaluate the stress tolerance of different crops and to identify tolerant genotypes due to involvement of multiple variables in its calculation [102, 103]. For instance, Chen et al. [42] used the membership function value for drought tolerance (MFVD) to identify superior genotypes in wheat. In the present study, we were able to identify 20 highly tolerant genotypes based on MFVP calculation. For example, GLKS 11,242 (Amanda), GLKS 10,800 (Limba), GLKS 38,155 (*S. chacoense*), GLKS 10,873 (Fransen), and GLKS 10,591 (Tiger) were among the best and were also found to be in the high-performing clusters identified by the heatmap based on standardized genotype means (Fig. 3, Table S1). Similarly, we were also able to identify the 20 worst performing or least tolerant genotypes based on the MFVP. Some of them, namely GLKS 11,578 (Kristall), GLKS 10,456 (Prudal) and GLKS 10,581 (Daresa), were also found in the worst performing cluster (Figs. 3 and 4) underpinning the usability of the MFVD.

### Genetic variation in the panel

P stress tolerance is a complex quantitative trait controlled by many genes and is strongly influenced by the environment [76, 104]. Thus, considerable genetic variation in a diversity panel and a high heritability are required for breeding and effective trait selection [102]. In our present study, broad sense heritability (H^2^_bs_) for the traits under study ranged from 33 to 88% and this showed substantial genetic contribution to the observed variance. Shoot biomass, root biomass, root length and plant height show moderate to high H^2^_bs_ values between 55 and 88%, which reflects a moderate to strong genetic influence. However, P utilization efficiency showed a lower heritability of 33%, suggesting predominant environmental influence on the trait. In general, our study included a diverse panel that exhibited considerable phenotypic variation across the traits of interest, which were found to be highly influenced by genetic factors. Thus, the considerable phenotypic variation, along with the reasonably high broad-sense heritability, suggest that the panel was suitable for conducting association studies on the accessions.

Furthermore, in the present study, we used genome-wide SNP markers to evaluate the genetic diversity of the panel of 200 potato accessions. The assessment of the relatedness performed by PCA and a model-based Bayesian population assignment provided strong evidence of distinct clusters, with cultivated GLKS accessions forming separate clusters from the wild species (Figs. 5a, 7 and 8). However, the *S. stenotomum* accession GLKS 24,129 clustered within the cultivated accessions (Figs. 5a and 8), which is supported by the ancestry proportions of GLKS 24,129 (Fig. 7). According to the GBIS database (GBIS: Gene Bank Information System; https://gbis.ipk-gatersleben.de/gbis2i), this accession originated from Bolivia as traditional cultivar/landrace. In the early stages of potato evolution in the northern Andes, diploid cultivated species from the *S. stenotomum* complex were likely selected from wild ancestors in the *S. brevicaule* complex, leading to the emergence of tetraploid *S. tuberosum* [105]. This suggests the possibility of the involvement of the accession GLKS 24,129 during domestication. The observed strong clustering of the older cultivated and the modern starch varieties could be advantageous in maintaining desired traits like high starch content, but potentially narrows the genetic base. Therefore, the inclusion of wild potato entries offers a potential to widen the genetic diversity.

### GWAS analysis for P efficiency in potato and identified marker-trait associations

Phosphorus efficiency is a complex trait, influenced by many genes that may exhibit additive, dominance and epistatic effects [106, 107]. Thus, it is important to dissect the genetic basis of the trait. Efforts have been made in the past towards the molecular dissections of P efficiency in various crops. Several QTL related to root, shoot and yield related traits have been reported in the past. Li et al. [108] detected QTL with additive and epistatic effects under different P supply conditions. They identified seven key candidate genes related to phosphate transporter or stress response for the QTLs detected under control or P deficiency conditions in maize [108]. Among the candidates were Phosphate transporter protein 1, AUXIN SIGNALING F-BOX 3, and genes related to root architecture [108]. Yan et al. [109] have recently mapped the QTL, *qBY3.1* to a region known to contain various P transporters of the PHT1 family. Various genes related to Pi signaling (PHR2) and Pi homeostasis (SPX3) were previously reported as candidates for QTL related to P efficiency [109–111].

We identified 27 MTAs linked to various phosphorus-related traits (Table 4). We found that the genotypes carrying two specific markers - loc1058_pos51 (associated with RFW_STI) and loc2624_pos27 (associated with ∆RFW), demonstrated enhanced root traits under LP conditions. Five genotype GLKS 11,422 (Erasme), GLKS 11,634 (Marfona), GLKS 11,723 (Paterson’s Victoria), GLKS 11,928 (Ulster Torch), and GLKS 24,129 (*S. stenotomum*) which exhibited increased root biomass under LP (Table S1) carried at least one of these SNPs (Additional file S2), with GLKS 11,634 (Marfona), GLKS 11,723 (Paterson’s Victoria) and GLKS 11,928 (Ulster Torch) harboring both. This suggests a potential cumulative effect of allelic variation at these loci in promoting root growth under phosphorus stress. The consistent presence of these SNPs in genotypes with superior root traits supports their putative functional role in phosphorus responsiveness. These findings underscore the value of these SNPs as promising molecular markers for use in marker-assisted selection to develop phosphorus-efficient potato cultivars.

Additionally, genotypes which exhibiting high P uptake under both HP and LP conditions i.e. GLKS 10,873 (Fransen), Standard 4, Standard 6, and Standrad 10 were found carry at least one of the markers loc642_pos101 (associated with PUE_LP and ∆PUE) and loc1290_pos62 (associated with PUE_STI) (Additional file S2). Thus, the presence of these markers in the P efficiency genotypes supports their potential functional relevance for P efficiency.

Similarly, high-biomass producing genotypes under both HP and LP conditions– GLKS 10,591 (Tiger), GLKS 10,800 (Limba), and GLKS 11,242 (Amanda) - were found to carry multiple SNPs associated with biomass traits (Additional file S2), including loc3882_pos47, loc8514_pos92, loc8926_pos11 (associated with SDW_STI), and loc2624_pos27 (associated with RFW_STI). The accumulation of these favorable alleles suggests a strong potential for enhanced biomass performance under LP conditions and underscores their breeding value for improving phosphorus efficiency through biomass-related traits.

In the present study, we were able to identify 22 putative candidate genes for significant marker trait associations of traits analyzed under HP and LP conditions (Tables 4 and 5). Two markers, loc642_pos101 and loc5675_pos10, were found to be significant for the traits ∆PUE and PUE under low P condition and for STIs for SDW, SFW, and TFW, respectively (Table 4). The difference in root fresh biomass showed the highest PVE (12.28%), suggesting a higher genetic control in this trait than the others. The traits SDW in HP, STI for PUE, and PH1 showed values near those found for the difference in root fresh biomass, that is, 11.12%, 11.03%, and 10.99%, respectively.

Among the 22 candidate genes, malate dehydrogenase (*Soltu.DM.01G031350*, MDH1), pectin methlyesterase (*Soltu.DM.01G031740*), F-box domain and WD40 domain protein (*Soltu.DM.01G032430*) and Myb-like DNA binding protein (*Soltu.DM.01G031190*) were associated with locus loc642_pos101 (PUE_LP, ∆PUE) (Table 5). Wang et al. [70] showed that the mitochondrial malate dehydrogenase gene isolated from *Gossypium hirsutum* L. (*GhmMDH1*) was involved in longer root growth under P deficiency in cotton. There was a significant decrease in biomass in the control wild type plants compared to *GHmMDH1* knockout plants [70]. Another putative gene encoding for pectin methylesterase (*Soltu.DM.01G031740*) was suggested to be involved in regulating P efficiency in soy [71]. The gene showed a significantly different expression under low P. Its expression was also reported to be root and stress specific [112].

Chen et al. [73] reported that a gene containing both WD40 and F-box motifs is a negative regulator of Pi starvation responses. Furthermore, a gene encoding for a Myb-like DNA binding protein was identified. Genes with Myb-DNA binding domains were previously found to be involved in the development of root architecture and in adaptation processes to P starvation [74, 113–115].

The locus loc1659_pos21, associated with PH1 under low P condition, was found to be located in a genomic region with various candidate genes like *Soltu.DM.02G018930*, belonging to the universal stress protein family (USP), Cytochrome P450 like protein (*Soltu.DM.02G019930*), and phosphofructokinase (*Soltu.DM.02G020660*, Tables 4 and 5). Yao et al. [75] found a significant upregulation of an USP related gene in the roots of low-P sensitive *Brassica napus* lines. Li et al. [76] reported that P450 genes were up-regulated in the roots of rice under low P. Iqbal et al. [79] demonstrated the enhanced activity of phosphofructokinase (PFK) in roots and shoots of cotton. They also reported an increase in the transcript level of PFK under low P in both roots and shoots.

The ABC transporter gene ABCG29 (*Soltu.DM.12GO10050*, Table 5) is a candidate found in the region of the association for SDW-STI on the marker loc8514_pos92 (Table 4). Aslam et al. [95] reported that the overexpression of ABCG29 of Lupin (*L. albABCG29*) in rice significantly improved P use under low P conditions through improved root growth and enhanced P accumulation in the cluster roots of the plant under low P conditions.

## Conclusion

The present study indicates a high phenotypic variation in our panel regarding the response to P availability. Many genotypes showed reduced growth under P deficient conditions, while adapting at the same time through increased root development and improved phosphorus utilization efficiency. The identification of high-performing accessions shows a potential to develop cultivars that are more efficient in P uptake and utilization, particularly for low-input agricultural systems. That could lead to the breeding of resilient nutrient-efficient potatoes. The results highlighted a significant genetic influence on some of the key traits like biomass, root length and P utilization efficiency. Furthermore, in the present study several significant loci were found to be associated with various phenotypic traits, identifying multiple putative candidate genes that could serve as suitable targets for breeding strategies.

## Supplementary Information

Supplementary Material 1.

Supplementary Material 2.

Supplementary Material 3.

Supplementary Material 4.

Supplementary Material 5.

## Data Availability

All data needed to evaluate the conclusions in the paper are included in the main part or supplementary material. Raw data can be obtained upon inquiry from the corresponding author.DNA sequence data for the accession are available in EMBL ESN database and can be accessed through via the link http://www.ebi.ac.uk/ena/data/view/PRJEB81675.We plan to make the original data of our study available on the e! DAL platform of the IPK (electronic data archive library, https://edal.ipk-gatersleben.de/) and the respective links can then be provided along with the revised manuscript.

## References

[1] Wishart J, George TS, Brown LK, Ramsay G, Bradshaw JE, White PJ, Gregory PJ. Measuring variation in potato roots in both field and glasshouse: the search for useful yield predictors and a simple screen for root traits. Plant Soil. 2013;368:231–49. 10.1007/s11104-012-1483-1.

[2] Thomas G, Sansonetti G. New light on a hidden treasure: International Year of the Potato 2008, an end-of-year review. Rome: Food and Agriculture Organization of the United Nations. 2009.

[3] Vilpoux OF, Junior JFSS. Global production and use of starch. In: Starchy crops morphology, extraction, properties and applications. Academic; 2023. p. 43–66. 10.1016/B978-0-323-90058-4.00014-1.

[4] Schumann P. Bedeutung der Kartoffel als nachwachsender Rohstoff. Kartoffelbau 67/8: 39-43 (DLG AgroFood medien gmbh, Gross-Umstadt). 2016.

[5] Tabata S, Hizukuri S. Studies on starch phosphate: part 2. Isolation of glucose 3-phosphate and maltose phosphate by acid hydrolysis of potato starch. Starch-Stärke. 1971;23:267–72. 10.1002/star.19710230803.

[6] Noda T, Kottearachchi NS, Tsuda S, Mori M, Takigawa S, Matsuura-Endo C, Kim S-J, Hashimoto N. Starch phosphorus content in potato (*Solanum tuberosum* L.) cultivars and its effect on other starch properties. Carbohydr Polym. 2007;68:793–6. 10.1016/j.carbpol.2006.08.005.

[7] Rosen CJ, Kelling KA, Stark JC, Porter GA. Optimizing phosphorus fertilizer management in potato production. Am J Potato Res. 2014;91:145–60. 10.1007/s12230-014-9371-2.

[8] Heinitz F, Farack K, Albert E. Verbesserung der P-Effizienz Im pflanzenbau. Schriftenreihe des landesamtes für umwelt, landwirtschaft und geologie. Dresden: Saxon State Office for Environment, Agriculture and Geology; 2013.

[9] Vance CP, Uhde-Stone C, Allan DL. Phosphorus acquisition and use: critical adaptations by plants for securing a nonrenewable resource. New Phytol. 2003;157:423–47. 10.1046/j.1469-8137.2003.00695.x. 33873400 10.1046/j.1469-8137.2003.00695.x

[10] Poirier Y, Bucher M. Phosphate transport and homeostasis in Arabidopsis. Arabidopsis Book. 2002;1: e0024. 10.1199/tab.0024. 22303200 10.1199/tab.0024 PMC3243343

[11] Hasan MM, Hasan MM, Teixeira da Silva JA, Li X. Regulation of phosphorus uptake and utilization: transitioning from current knowledge to practical strategies. Cell Mol Biol Lett. 2016;21:7. 10.1186/s11658-016-0008-y. 28536610 10.1186/s11658-016-0008-y PMC5415736

[12] Shen J, Yuan L, Zhang J, Li H, Bai Z, Chen X, Zhang W, Zhang F. Phosphorus dynamics: from soil to plant. Plant Physiol. 2011;156:997–1005. 10.1104/pp.111.175232. 21571668 10.1104/pp.111.175232 PMC3135930

[13] Weaver JE. Root development of field crops. In: Piper CV, editor. McGraw-Hill publications in the agricultural and biological sciences. New York: McGraw-Hill Book Co., Inc.; 1926. p. 291.

[14] Fixen PE, Bruulsema TW. Potato management challenges created by phosphorus chemistry and plant roots. Am J Potato Res. 2014;91(2):121–31. 10.1007/s12230-014-9374-z.

[15] Hopkins BG, Horneck DA, MacGuidwin AE. Improving phosphorus use efficiency through potato rhizosphere modification and extension. Am J Potato Res. 2014;91:161–74. 10.1007/s12230-014-9370-3.

[16] Ha S, Tran L-S. Understanding plant responses to phosphorus starvation for improvement of plant tolerance to phosphorus deficiency by biotechnological approaches. Crit Rev Biotechnol. 2014;34:16–30. 10.3109/07388551.2013.783549. 23586682 10.3109/07388551.2013.783549

[17] López-Arredondo DL, Leyva-González MA, González-Morales SI, López-Bucio J, Herrera-Estrella L. Phosphate nutrition: improving low-phosphate tolerance in crops. Annu Rev Plant Biol. 2014;65:95–123. 10.1146/annurev-arplant-050213-035949. 24579991 10.1146/annurev-arplant-050213-035949

[18] Xiao M, Burford MA, Wood SA, Aubriot L, Ibelings BW, Prentice MJ, Galvanese EF, Harris TD, Hamilton DP. Schindler’s legacy: from eutrophic lakes to the phosphorus utilization strategies of cyanobacteria. FEMS Microbiol Rev. 2022. 10.1093/femsre/fuac029. 10.1093/femsre/fuac029 PMC9629505 35749580

[19] Dhillon J, Torres G, Driver E, Figueiredo B, Raun WR. World phosphorus use efficiency in cereal crops. Agron J. 2017;109:1670–7. 10.2134/agronj2016.08.0483.

[20] Sattari SZ, Bouwman AF, Giller KE, van Ittersum MK. Residual soil phosphorus as the missing piece in the global phosphorus crisis puzzle. Proc Natl Acad Sci. 2012;109:6348–53. 10.1073/pnas.1113675109. 22431593 10.1073/pnas.1113675109 PMC3341047

[21] Richardson AE, Lynch JP, Ryan PR, Delhaize E, Smith FA, Smith SE, Harvey PR, Ryan MH, Veneklaas EJ, Lambers H, Oberson A, Culvenor RA, Simpson RJ. Plant and microbial strategies to improve the phosphorus efficiency of agriculture. Plant Soil. 2011;349:121–56. 10.1007/s11104-011-0950-4.

[22] Hinsinger P. Bioavailability of soil inorganic P in the rhizosphere as affected by root-induced chemical changes: a review. Plant Soil. 2001;237:173–95. 10.1023/A:1013351617532.

[23] Fernandes AM, Soratto RP, Gonsales JR. Root morphology and phosphorus uptake by potato cultivars grown under deficient and sufficient phosphorus supply. Sci Hortic. 2014;180:190–8. 10.1016/j.scienta.2014.10.035.

[24] Schenk MK, Barber SA. Potassium and phosphorus uptake by corn genotypes grown in the field as influenced by root characteristics. Plant Soil. 1980;54:65–76. 10.1007/bf02182000.

[25] Wacker-Fester K, Uptmoor R, Pfahler V, Dehmer KJ, Bachmann-Pfabe S, Kavka M. Genotype-specific differences in phosphorus efficiency of potato (*Solanum tuberosum* L). Front Plant Sci. 2019;10:1029. 10.3389/fpls.2019.01029. 31475025 10.3389/fpls.2019.01029 PMC6706458

[26] Kirchgesser J, Hazarika M, Bachmann-Pfabe S, Dehmer KJ, Kavka M, Uptmoor R. Phenotypic variation of root-system architecture under high P and low P conditions in potato (*Solanum tuberosum* L). BMC Plant Biol. 2023;23:68. 10.1186/s12870-023-04070-9. 36721096 10.1186/s12870-023-04070-9 PMC9890858

[27] Liu F, Chang X-J, Ye Y, Xie W-B, Wu P, Lian X-M. Comprehensive sequence and whole-life-cycle expression profile analysis of the phosphate transporter gene family in rice. Mol Plant. 2011;4:1105–22. 10.1093/mp/ssr058. 21832284 10.1093/mp/ssr058

[28] Miller SS, Liu J, Allan DL, Menzhuber CJ, Fedorova M, Vance CP. Molecular control of acid phosphatase secretion into the rhizosphere of proteoid roots from phosphorus-stressed white lupin. Plant Physiol. 2001;127:594–606. 10.1104/pp.010097. 11598233 PMC125094

[29] Kavka M, Korn K, Hazarika M, Bachmann-Pfabe S, Uptmoor R. Potato root and leaf phosphatase activity in response to P deprivation. J Plant Nutr Soil Sci. 2021;184:668–77. 10.1002/jpln.202100112.

[30] Grierson CS, Parker JS, Kemp AC. Arabidopsis genes with roles in root hair development. J Plant Nutr Soil Sci. 2001;164:131–40.

[31] Raghothama KG. Phosphate acquisition. Annu Rev Plant Physiol Plant Mol Biol. 1999;50:665–93. 10.1146/annurev.arplant.50.1.665. 15012223 10.1146/annurev.arplant.50.1.665

[32] Zhang H, Forde BG. An Arabidopsis MADS box gene that controls nutrient-induced changes in root architecture. Science. 1998;279:407–9. 10.1126/science.279.5349.407. 9430595 10.1126/science.279.5349.407

[33] Cho H-T, Cosgrove DJ. Regulation of root hair initiation and expansin gene expression in Arabidopsis. Plant Cell. 2002;14:3237–53. 10.1105/tpc.006437. 12468740 10.1105/tpc.006437 PMC151215

[34] Uhde-Stone C, Zinn KE, Ramirez-Yáñez M, Li A, Vance CP, Allan DL. Nylon filter arrays reveal differential gene expression in proteoid roots of white lupin in response to phosphorus deficiency. Plant Physiol. 2003;131:1064–79. 10.1104/pp.102.016881. 12644659 10.1104/pp.102.016881 PMC166872

[35] Hirschhorn JN, Daly MJ. Genome-wide association studies for common diseases and complex traits. Nat Rev Genet. 2005;6:95–108. 10.1038/nrg1521. 15716906 10.1038/nrg1521

[36] Wang C, Yang Y, Yuan X, Xu Q, Feng Y, Yu H, Wang Y. Genome-wide association study of blast resistance in *indica* rice. BMC Plant Biol. 2014;14:311. 10.1186/s12870-014-0311-6. 25403621 10.1186/s12870-014-0311-6 PMC4239320

[37] Hammond JP, Broadley MR, Bowen HC, Spracklen WP, Hayden RM, White PJ. Gene expression changes in phosphorus deficient potato (*Solanum tuberosum* L.) leaves and the potential for diagnostic gene expression markers. PLoS One. 2011;6:e24606. 10.1371/journal.pone.0024606. 21935429 10.1371/journal.pone.0024606 PMC3173461

[38] Murashige T, Skoog F. A revised medium for rapid growth and bio assays with tobacco tissue cultures. Physiol Plant. 1962;15:473–97. 10.1111/j.1399-3054.1962.tb08052.x.

[39] Hoagland DR, Aron DI. Nutrient solutions for hydroponic culture. Univ Calif Agricultural Experimental Stn Circular. 1938;347.

[40] Page AL, Miller RH, Keeney DR. Methods of soil analysis: part 2. Chemical and microbiological properties. Madison, WI, USA: Soil Science Society of America; 1982.

[41] Fernandez GCJ. Effective selection criteria for assessing plant stress tolerance. In: Kuo CG, editor. Adaptation of food crops to temperature and water stress: proceedings of an international symposium, Taiwan, 13–18 August 1992. Taipei: AVRDC; 1993. p. 257–270.

[42] Chen X, Min D, Yasir TA, Hu Y-G. Evaluation of 14 morphological, yield-related and physiological traits as indicators of drought tolerance in Chinese winter bread wheat revealed by analysis of the membership function value of drought tolerance (MFVD). Field Crops Res. 2012;137:195–201. 10.1016/j.fcr.2012.09.008.

[43] R Core Team. R: A Language and environment for statistical computing 2022. Vienna, Austria. https://www.R-project.org/.

[44] Rosmaina R, Syafrudin S, Hasrol H, Yanti F, Juliyanti J, Zulfahmi Z. Estimation of variability, heritability and genetic advance among local chili pepper genotypes cultivated in peat lands. Bulg J Agric Sci. 2016;22:431–6.

[45] Toker C. Estimates of broad-sense heritability for seed yield and yield criteria in faba bean (*Vicia faba* L). Hereditas. 2004;140:222–5. 10.1111/j.1601-5223.2004.01780.x. 15198712 10.1111/j.1601-5223.2004.01780.x

[46] Galili T, O’Callaghan A, Sidi J, Sievert C. Heatmaply: an R package for creating interactive cluster heatmaps for online publishing. Bioinformatics. 2018;34:1600–2. 10.1093/bioinformatics/btx657. 29069305 10.1093/bioinformatics/btx657 PMC5925766

[47] Doyle JJ, Doyle JL. Isolation of plant DNA from fresh tissue. Focus. 1990;12:13–5.

[48] Elshire RJ, Glaubitz JC, Sun Q, Poland JA, Kawamoto K, Buckler ES, Mitchell SE. A robust, simple genotyping-by-sequencing (GBS) approach for high diversity species. PLoS One. 2011;6:e19379. 10.1371/journal.pone.0019379. 21573248 10.1371/journal.pone.0019379 PMC3087801

[49] Wendler N, Mascher M, Nöh C, Himmelbach A, Scholz U, Ruge-Wehling B, Stein N. Unlocking the secondary gene-pool of barley with next-generation sequencing. Plant Biotechnol J. 2014;12:1122–31. 10.1111/pbi.12219. 25040223 10.1111/pbi.12219

[50] Andrews S. FastQC: A quality control tool for high throughput sequence data. 2010. http://www.bioinformatics.babraham.ac.uk/projects/fastqc/.

[51] Martin M. Cutadapt removes adapter sequences from high-throughput sequencing reads. EMBnet J. 2011;17: 10. 10.14806/ej.17.1.200.

[52] Eaton DAR, Overcast I. Ipyrad: interactive assembly and analysis of RADseq datasets. Bioinformatics. 2020;36:2592–4. 10.1093/bioinformatics/btz966. 31904816 10.1093/bioinformatics/btz966

[53] Eaton DAR, PyRAD. Assembly of de Novo RADseq loci for phylogenetic analyses. Bioinformatics. 2014;30:1844–9. 10.1093/bioinformatics/btu121. 24603985 10.1093/bioinformatics/btu121

[54] Frichot E, François O. LEA: an R package for landscape and ecological association studies. Methods Ecol Evol. 2015;6:925–9. 10.1111/2041-210x.12382.

[55] Wickham H, Averick M, Bryan J, Chang W, McGowan L, François R, Grolemund G, Hayes A, Henry L, Hester J, Kuhn M, Pedersen T, Miller E, Bache S, Müller K, Ooms J, Robinson D, Seidel D, Spinu V, Takahashi K, Vaughan D, Wilke C, Woo K, Yutani H. Welcome to the tidyverse. JOSS. 2019;4:1686. 10.21105/joss.01686.

[56] Wickham H. Data Analysis. In: Wickham H, editor. ggplot2. Springer International Publishing; 2016. p. 189–201. 10.1007/978-3-319-24277-4_9.

[57] Nguyen L-T, Schmidt HA, von Haeseler A, Minh BQ. IQ-tree: a fast and effective stochastic algorithm for estimating maximum-likelihood phylogenies. Mol Biol Evol. 2015;32:268–74. 10.1093/molbev/msu300. 25371430 10.1093/molbev/msu300 PMC4271533

[58] Minh BQ, Schmidt HA, Chernomor O, Schrempf D, Woodhams MD, von Haeseler A, Lanfear R. IQ-tree 2: new models and efficient methods for phylogenetic inference in the genomic era. Mol Biol Evol. 2020;37:1530–4. 10.1093/molbev/msaa015. 32011700 10.1093/molbev/msaa015 PMC7182206

[59] Danecek P, Auton A, Abecasis G, Albers CA, Banks E, DePristo MA, Handsaker RE, Lunter G, Marth GT, Sherry ST, McVean G, Durbin R. The variant call format and vcftools. Bioinformatics. 2011;27:2156–8. 10.1093/bioinformatics/btr330. 21653522 10.1093/bioinformatics/btr330 PMC3137218

[60] Ortiz EM. vcf2phylip: convert a VCF matrix into several matrix formats for phylogenetic analysis. Zenodo; 2019. 10.5281/zenodo.2540861.

[61] Kalyaanamoorthy S, Minh BQ, Wong TKF, von Haeseler A, Jermiin LS. Modelfinder: fast model selection for accurate phylogenetic estimates. Nat Methods. 2017;14:587–9. 10.1038/nmeth.4285. 28481363 10.1038/nmeth.4285 PMC5453245

[62] Hoang DT, Chernomor O, von Haeseler A, Minh BQ, Le Vinh S. UFBoot2: improving the ultrafast bootstrap approximation. Mol Biol Evol. 2018;35:518–22. 10.1093/molbev/msx281. 29077904 10.1093/molbev/msx281 PMC5850222

[63] Purcell S, Neale B, Todd-Brown K, Thomas L, Ferreira MAR, Bender D, et al. PLINK: a tool set for whole-genome association and population-based linkage analyses. Am J Hum Genet. 2007;81:559–75. 10.1086/519795. 17701901 10.1086/519795 PMC1950838

[64] Yin L, Zhang H, Tang Z, Xu J, Yin D, Zhang Z, Yuan X, Zhu M, Zhao S, Li X, Liu. A Memory-efficient, Visualization-enhanced, and Parallel-accelerated tool for Genome-wide association study. Genomics Proteom Bioinf. 2021;19:619–28. 10.1016/j.gpb.2020.10.007. 10.1016/j.gpb.2020.10.007 PMC9040015 33662620

[65] Liu X, Huang M, Fan B, Buckler ES, Zhang Z. Iterative usage of fixed and random effect models for powerful and efficient genome-wide association studies. PLoS Genet. 2016;12:e1005767. 10.1371/journal.pgen.1005767. 26828793 10.1371/journal.pgen.1005767 PMC4734661

[66] Goodstein DM, Shu S, Howson R, Neupane R, Hayes RD, Fazo J, Mitros T, Dirks W, Hellsten U, Putnam N. 826 Rokhsar DS. Phytozome: A comparative platform for green plant genomics. Nucleic Acids Res. 2012;40:D1178–86. 10.1093/nar/gkr944. 22110026 10.1093/nar/gkr944 PMC3245001

[67] Shim H, Chasman DI, Smith JD, Mora S, Ridker PM, Nickerson DA, Krauss RM, Stephens M. A multivariate genome-wide association analysis of 10 LDL subfractions, and their response to statin treatment, in 1868 Caucasians. PLoS One. 2015;10:e0120758. 10.1371/journal.pone.0120758. 25898129 10.1371/journal.pone.0120758 PMC4405269

[68] Alexander DH, Lange K. Enhancements to the ADMIXTURE algorithm for individual ancestry estimation. BMC Bioinformatics. 2011;12:246. 10.1186/1471-2105-12-246. 21682921 10.1186/1471-2105-12-246 PMC3146885

[69] Frichot E, Mathieu F, Trouillon T, Bouchard G, François O. Fast and efficient estimation of individual ancestry coefficients. Genetics. 2014;196:973–83. 10.1534/genetics.113.160572. 24496008 10.1534/genetics.113.160572 PMC3982712

[70] Wang Z-A, Li Q, Ge X-Y, Yang C-L, Luo X-L, Zhang A-H, Xiao J-L, Tian Y-C, Xia G-X, Chen X-Y, Li F-G, Wu J-H. The mitochondrial malate dehydrogenase 1 gene *GhmMDH1* is involved in plant and root growth under phosphorus deficiency conditions in cotton. Sci Rep. 2015;5:10343. 10.1038/srep10343. 26179843 10.1038/srep10343 PMC4503954

[71] Zhang D, Li H, Wang J, Zhang H, Hu Z, Chu S, Lv H, Yu D. High-density genetic mapping identifies new major loci for tolerance to low-phosphorus stress in soybean. Front Plant Sci. 2016;7:372. 10.3389/fpls.2016.00372. 27065041 10.3389/fpls.2016.00372 PMC4811872

[72] Akash, Parida AP, Srivastava A, Mathur S, Sharma AK, Kumar R. Identification, evolutionary profiling, and expression analysis of F-box superfamily genes under phosphate deficiency in tomato. Plant Physiol Biochem. 2021;162:349–62. 10.1016/j.plaphy.2021.03.002. 33730620 10.1016/j.plaphy.2021.03.002

[73] Chen Z-H, Jenkins GI, Nimmo HG. Identification of an F-box protein that negatively regulates Pi starvation responses. Plant Cell Physiol. 2008;49:1902–6. 10.1093/pcp/pcn157. 18930958 10.1093/pcp/pcn157

[74] Reddy VRP, Das S, Dikshit HK, Mishra GP, Aski M, Meena SK, Singh A, Pandey R, Singh MP, Tripathi K, Gore PG, Priti N, Bhagat TK, Kumar S, Nair R, Sharma TR. Genome-Wide association analysis for phosphorus use efficiency traits in Mungbean (*Vigna radiata* L. Wilczek) using genotyping by sequencing approach. Front Plant Sci. 2020;11:537766. 10.3389/fpls.2020.537766. 33193476 10.3389/fpls.2020.537766 PMC7658405

[75] Yao Y, Sun H, Xu F, Zhang X, Liu S. Comparative proteome analysis of metabolic changes by low phosphorus stress in two *Brassica napus* genotypes. Planta. 2011;233:523–37. 10.1007/s00425-010-1311-x. 21110039 10.1007/s00425-010-1311-x

[76] Li L, Liu C, Lian X. Gene expression profiles in rice roots under low phosphorus stress. Plant Mol Biol. 2010;72:423–32. 10.1007/s11103-009-9580-0. 19936943 10.1007/s11103-009-9580-0

[77] Lin S, Yu L, Zhang H. Transcriptomic responses to thermal stress and varied phosphorus conditions in *Fugacium Kawagutii*. Microorganisms. 2019. 10.3390/microorganisms7040096. 10.3390/microorganisms7040096 PMC6517890 30987028

[78] Guo X, Ullah A, Siuta D, Kukfisz B, Iqbal S. Role of WRKY transcription factors in regulation of abiotic stress responses in cotton. Life. 2022; 1410. 10.3390/life12091410. 36143446 10.3390/life12091410 PMC9504182

[79] Iqbal A, Qiang D, Xiangru W, Huiping G, Hengheng Z, Xiling Z, Meizhen S. Phosphorus and carbohydrate metabolism contributes to low phosphorus tolerance in cotton. BMC Plant Biol. 2023;23:97. 10.1186/s12870-023-04100-6. 10.1186/s12870-023-04100-6 PMC9933316 36792994

[80] Wang J, Pan W, Nikiforov A, King W, Hong W, Li W, Han Y, Patton-Vogt J, Shen J, Cheng L. Identification of two glycerophosphodiester phosphodiesterase genes in maize leaf phosphorus remobilization. Crop J. 2021;9:95–108. 10.1016/j.cj.2020.05.004. 40735295 10.1016/j.cj.2020.05.004 PMC12306635

[81] Srivastava S, Kaur J, Anand V, Singh VB, Singh P, Rastogi S, Yadav S, Srivastava S. Phosphate starvation responses in plants and microbe mediated phosphorus recycling in soil: a review. Int J Plant Anim Environ Sci. 2022;8:25–37. 10.18811/ijpen.v8i01.03.

[82] McCarthy L, Abramchuk I, Wafy G, Denoncourt A, Lavallée-Adam M, Downey M. Ddp1 cooperates with Ppx1 to counter a stress response initiated by nonvacuolar polyphosphate. mBio. 2022;13:e0039022. 10.1128/mbio.00390-22. 35862758 10.1128/mbio.00390-22 PMC9426566

[83] Gu M, Chen A, Sun S, Xu G. Complex regulation of plant phosphate transporters and the gap between molecular mechanisms and practical application: what is missing? Mol Plant. 2016;9:396–416. 26714050 10.1016/j.molp.2015.12.012

[84] Zhang Z, Liao H, Lucas WJ. Molecular mechanisms underlying phosphate sensing, signaling, and adaptation in plants. J Integr Plant Biol. 2014;56:192–220. 10.1111/jipb.12163. 24417933 10.1111/jipb.12163

[85] Cai Y, Qi J, Li C, Miao K, Jiang B, Yang X, Han W, Wang Y, Gao J, Dong X. Genome-wide analysis of purple acid phosphatase genes in *Brassica rapa* and their association with pollen development and phosphorus deprivation stress. Horticulturae. 2021;7:363. 10.3390/horticulturae7100363.

[86] Zimmermann P. Root-secreted phosphomonoesterases mobilizing phosphorus from the rhizosphere: A molecular physiological study in Solanum tuberosum. Zurich: ETH; 2003.

[87] Fan F, Wang Q, Wen X, Ding G. Transcriptome-wide identification and expression profiling of *Pinus massoniana* MYB transcription factors responding to phosphorus deficiency. J Res. 2020;31:909–19. 10.1007/s11676-019-00911-2.

[88] Gutiérrez-Luna FM, Hernández-Domínguez EE, Valencia-Turcotte LG, Rodríguez-Sotres R. Review. Pyrophosphate and pyrophosphatases in plants, their involvement in stress responses and their possible relationship to secondary metabolism. Plant Sci. 2018;267:11–9. 10.1016/j.plantsci.2017.10.016. 29362089 10.1016/j.plantsci.2017.10.016

[89] Wen Y, Li X, Guo C, Ma C, Duan W, Lu W, Xiao K. Characterization and expression analysis of mitogen-activated protein kinase cascade genes in wheat subjected to phosphorus and nitrogen deprivation, high salinity, and drought. J Plant Biochem Biotechnol. 2015;24:184–96. 10.1007/s13562-014-0256-8.

[90] Kumar A, Gahlaut V, Nagaraju M. Transcription factors and their roles in phosphorus stress tolerance in crop plants. Transcription factors for abiotic stress tolerance in plants. Academic; 2020. pp. 201–24. 10.1016/B978-0-12-819334-1.00011-3.

[91] Fang W, Ding W, Zhao X, Zhang F, Gao S, Li X, Xiao K. Expression profile and function characterization of the MYB type transcription factor genes in wheat (*Triticum aestivum* L.) under phosphorus deprivation. Acta Physiol Plant. 2016;38:63. 10.1007/s11738-015-1997-2.

[92] Shi S-Y, Zhang F-F, Gao S, Xiao K. Expression pattern and function analyses of the MADS transcription factor genes in wheat (*Triticum aestivum* L.) under phosphorus-starvation condition. J Integr Agric. 2016;15:1703–15. 10.1016/s2095-3119(15)61167-4.

[93] Ma J, Gao S, Jiang Q-T, Yang Q, Sun M, Wang J-R, Qi P-F, Liu Y-X, Li W, Pu Z-E, Lan X-J, Wei Y-M, Liu C, Zheng Y-L. Structure and expression of phosphoglucan phosphatase genes of *Like sex Four1* and *Like sex Four2* in barley. Genetica. 2016;144:313–23. 10.1007/s10709-016-9900-7. 27154345 10.1007/s10709-016-9900-7

[94] Chen Y-F, Li L-Q, Xu Q, Kong Y-H, Wang H, Wu W-H. The WRKY6 transcription factor modulates *PHOSPHATE1* expression in response to low Pi stress in *Arabidopsis*. Plant Cell. 2009;21:3554–66. 10.1105/tpc.108.064980. 10.1105/tpc.108.064980 PMC2798333 19934380

[95] Aslam MM, Waseem M, Zhang Q, Ke W, Zhang J, Xu W. Identification of ABC transporter G subfamily in white lupin and functional characterization of *L. albABGC29* in phosphorus use. BMC Genomics. 2021;22:723. 10.1186/s12864-021-08015-0. 34615466 10.1186/s12864-021-08015-0 PMC8495970

[96] Narang RA, Bruene A, Altmann T. Analysis of phosphate acquisition efficiency in different Arabidopsis accessions. Plant Physiol. 2000;124:1786–99. 10.1104/pp.124.4.1786. 11115894 10.1104/pp.124.4.1786 PMC59875

[97] Schenk MK. Nutrient efficiency of vegetable crops. Acta Hort. 2006;21–34. 10.17660/actahortic.2006.700.1.

[98] Rose TJ, Rose MT, Pariasca-Tanaka J, Heuer S, Wissuwa M. The frustration with utilization: why have improvements in internal phosphorus utilization efficiency in crops remained so elusive?? Front Plant Sci. 2011;2: 73. 10.3389/fpls.2011.00073. 22639608 10.3389/fpls.2011.00073 PMC3355673

[99] Bayuelo-Jiménez JS, Ochoa-Cadavid I. Phosphorus acquisition and internal utilization efficiency among maize landraces from the central Mexican highlands. Field Crops Res. 2014;156:123–34. 10.1016/j.fcr.2013.11.005.

[100] Gemenet DC, Hash CT, Sanogo MD, Sy O, Zangre RG, Leiser WL, Haussmann BIG. Phosphorus uptake and utilization efficiency in West African pearl millet inbred lines. Field Crops Res. 2015;171:54–66. 10.1016/j.fcr.2014.11.001.

[101] Bachmann-Pfabe S, Dehmer KJ. Evaluation of wild potato germplasm for tuber starch content and nitrogen utilization efficiency. Plants. 2020; 833. 10.3390/plants9070833. 32630783 10.3390/plants9070833 PMC7411790

[102] Yan C, Song S, Wang W, Wang C, Li H, Wang F, Li S, Sun X. Screening diverse soybean genotypes for drought tolerance by membership function value based on multiple traits and drought-tolerant coefficient of yield. BMC Plant Biol. 2020;20:321. 10.1186/s12870-020-02519-9. 32640999 10.1186/s12870-020-02519-9 PMC7346468

[103] Pathania S, Singh H. Evaluation and prediction of salinity tolerance behavior of citrus rootstocks. Sci Hortic. 2021;289: 110422. 10.1016/j.scienta.2021.110422.

[104] Deng Q, Dai L, Chen Y, Wu D, Shen Y, Xie J, Luo X. Identification of phosphorus stress related proteins in the seedlings of Dongxiang wild rice (*Oryza rufipogon* Griff.) using label-free quantitative proteomic analysis. Genes. 2022;13:108. 10.3390/genes13010108. 35052448 10.3390/genes13010108 PMC8774503

[105] Grun P. The evolution of cultivated potatoes. Econ Bot. 1990;44:39–55. 10.1007/bf02860474.

[106] Duncan RR, Carrow RN. Turfgrass molecular genetic improvement for abiotic/edaphic stress resistance. Adv Agron. 1999;67:233–305.

[107] Shenoy VV, Kalagudi GM. Enhancing plant phosphorus use efficiency for sustainable cropping. Biotechnol Adv. 2005;23:501–13. 10.1016/j.biotechadv.2005.01.004. 16140488 10.1016/j.biotechadv.2005.01.004

[108] Li D, Wang H, Wang M, Li G, Chen Z, Leiser WL, Weiß TM, Lu X, Wang M, Chen S, Chen F, Yuan L, Würschum T, Liu W. Genetic dissection of phosphorus use efficiency in a maize association population under two P levels in the field. Int J Mol Sci. 2021;22: 9311. 10.3390/ijms22179311. 34502218 10.3390/ijms22179311 PMC8430673

[109] Yan M, Feng F, Xu X, Fan P, Lou Q, Chen L, et al. Genome-wide association study identifies a gene conferring high physiological phosphorus use efficiency in rice. Front Plant Sci. 2023;14:1153967. 10.3389/fpls.2023.1153967. 36998687 10.3389/fpls.2023.1153967 PMC10043302

[110] Zhou J, Jiao F, Wu Z, Li Y, Wang X, He X, et al. *OsPHR2* is involved in phosphate-starvation signaling and excessive phosphate accumulation in shoots of plants. Plant Physiol. 2008;146:1673–86. 10.1104/pp.107.111443. 18263782 10.1104/pp.107.111443 PMC2287342

[111] Shi J, Hu H, Zhang K, Zhang W, Yu Y, Wu Z, Wu P. The paralogous *SPX3* and *SPX5* genes redundantly modulate Pi homeostasis in rice. J Exp Bot. 2014;65:859–70. 10.1093/jxb/ert424. 24368504 10.1093/jxb/ert424 PMC3924727

[112] Pelloux J, Rustérucci C, Mellerowicz EJ. New insights into pectin methylesterase structure and function. Trends Plant Sci. 2007;12(6):267–77. 10.1016/j.tplants.2007.04.001. 17499007 10.1016/j.tplants.2007.04.001

[113] Devaiah BN, Madhuvanthi R, Karthikeyan AS, Raghothama KG. Phosphate starvation responses and gibberellic acid biosynthesis are regulated by the MYB62 transcription factor in Arabidopsis. Mol Plant. 2009;2:43–58. 10.1093/mp/ssn081. 19529828 10.1093/mp/ssn081 PMC2639739

[114] Dai X, Wang Y, Yang A, Zhang W-H. *OsMYB2P-1*, an R2R3 MYB transcription factor, is involved in the regulation of phosphate-starvation responses and root architecture in rice. Plant Physiol. 2012;159:169–83. 10.1104/pp.112.194217. 22395576 10.1104/pp.112.194217 PMC3375959

[115] Baek D, Kim MC, Chun HJ, Kang S, Park HC, Shin G, Park J, Shen M, Hong H, Kim W-Y, Kim DH, Lee SY, Bressan RA, Bohnert HJ, Yun D-J. Regulation of *miR399f* transcription by AtMYB2 affects phosphate starvation responses in Arabidopsis. Plant Physiol. 2013;161:362–73. 10.1104/pp.112.205922. 23154535 10.1104/pp.112.205922 PMC3532267

