# Supplementary material for: Phenotypic variability and genome-wide association studies in potato (Solanum tuberosum L.) for phosphorus efficiency
Source: BMC Plant Biol. 2025 Aug 2;25:1012. doi: 10.1186/s12870-025-07018-3 (PMC12317545; doi:10.1186/s12870-025-07018-3)
Supplement: Supplementary file 3 — Supplementary Material 3. [file 12870_2025_7018_MOESM3_ESM.pdf]

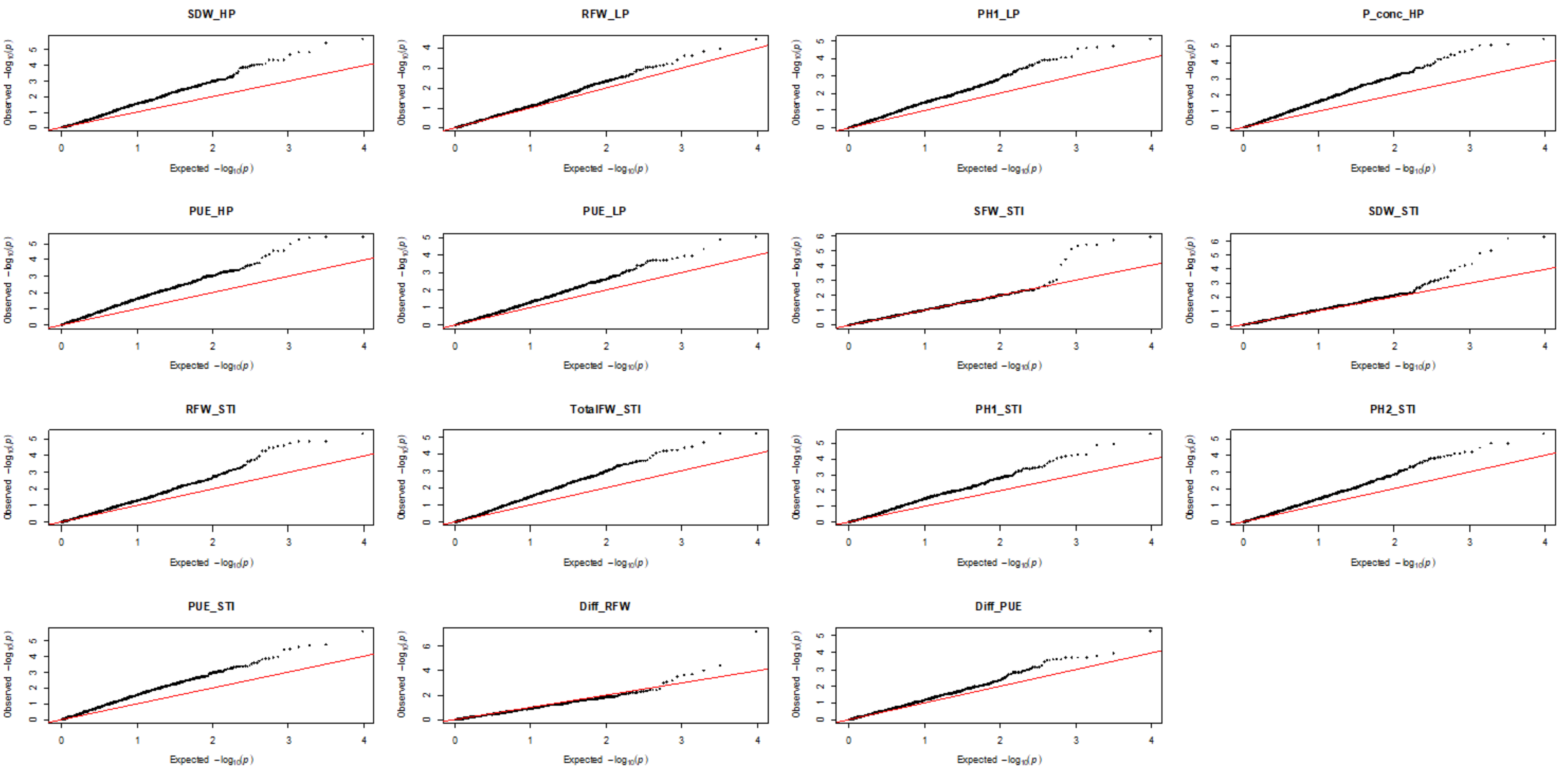

Fig. S2: Quantile-quantile plots illustrating the comparison between expected and observed -log1o(p)-values to detect significant MTas associated with the phenotypic traits : shoot dry weight under high P (SDW_HP); root fresh weight under low P (RFW_LP); plant height after 1 week under low (PH1_LP); phosphorus concentration under high P (P_conc_HP); phosphorus utilisation efficiency under high and low P (PUE_HP, PUE_LP); stress tolerance indices for shoot fresh weight (SFW_STI), shoot dry weight (SDW_STI), root fresh weight (RFW_STI), total fresh weight (TotalFW_STI), plant height after 1 week (PH1_STI), plant height after 4 weeks (PH2_STI) and phosphorus utilization efficiency (PUE_STI); difference between root fresh weight under high and low P (Diff_RFW) and difference between phosphorus utilisation efficiency under high and low P (Diff_PUE). The blue horizontal line indicates the threshold level for significant p [$-\log_{10}(p) = 5$]
